# Structural heterogeneities in starch hydrogels

**DOI:** 10.1016/j.carbpol.2020.116834

**Published:** 2020-12-01

**Authors:** Todor T. Koev, Juan C. Muñoz-García, Dinu Iuga, Yaroslav Z. Khimyak, Frederick J. Warren

**Affiliations:** a School of Pharmacy, University of East Anglia, Norwich Research Park, NR4 7TJ, UK; b Food Innovation and Health, Quadram Institute Bioscience, Norwich Research Park, NR4 7UQ, UK; c Department of Physics, University of Warwick, Coventry CV4 7AL, UK

**Keywords:** NMR, nuclear magnetic resonance, CP/MAS, cross polarisation – magic angle spinning, CPSP/MAS, cross polarisation single pulse – magic angle spinning, HR-MAS, high-resolution – magic angle spinning, WPTCP, water polarisation transfer cross polarisation, DSC, differential scanning calorimetry, PXRD, powder x-ray diffraction, Starch hydrogels, NMR spectroscopy, CP/MAS NMR, CPSP/MAS NMR, Network organisation, Internal dynamics

## Abstract

Hydrogels have a complex, heterogeneous structure and organisation, making them promising candidates for advanced structural and cosmetics applications. Starch is an attractive material for producing hydrogels due to its low cost and biocompatibility, but the structural dynamics of polymer chains within starch hydrogels are not well understood, limiting their development and utilisation. We employed a range of NMR methodologies (CPSP/MAS, HR-MAS, HPDEC and WPT-CP) to probe the molecular mobility and water dynamics within starch hydrogels featuring a wide range of physical properties. The insights from these methods were related to bulk rheological, thermal (DSC) and crystalline (PXRD) properties. We have reported for the first time the presence of highly dynamic starch chains, behaving as solvated moieties existing in the liquid component of hydrogel systems. We have correlated the chains’ degree of structural mobility with macroscopic properties of the bulk systems, providing new insights into the structure-function relationships governing hydrogel assemblies.

## Highlights

• Produced starch hydrogels through high temperature-pressure gelatinisation.
• Employed a range of NMR methods to probe the molecular mobility and water dynamics.
• Reported for the first time highly dynamic starch chains in the solvent phase of gels.
• Correlated the degree of chain structural mobility with bulk properties.
• Revealed a previously unknown level of molecular organisation in starch gels.

## 1 Introduction

Hydrogels are a group of materials comprised of cross-linked hydrophilic polymers forming a large network structure, enabling them to hold large amounts of water and/or other biological fluids within their three-dimensional network (Spagnol et al., 2012). Properties, such as their hydrophilicity, low interfacial tension, adaptability/mouldability, swelling and capillary properties and environmental responsivity, have earned them their place as highly promising candidates for the development of biocompatible scaffolds, implants and “smart” drug delivery materials. A wide range of both natural (*e.g.* starch, pectin, cellulose, chitosan) and synthetic (*e.g.* poly(vinyl alcohol), poly(*N*-vinyl pyrrolidone)) hydrogels are employed in a great number of industrial, pharmaceutical and environmental spheres (Chang, Duan, Cai, & Zhang, 2010; Peppas, Bures, Leobandung, & Ichikawa, 2000).

Starch is a naturally occurring polymer made up of two high molecular weight, polydisperse [1→4]-α-D-glucose polysaccharides – amylose and amylopectin. Amylose is a predominantly linear polysaccharide featuring mainly [1→4]-α bonds and very few [1→6]-α branching points. Its long linear structure allows for inter-glucan interactions, such as entangling and proximal alignment. In contrast, amylopectin features multiple [1→6]-α linkages, resulting in a highly branched structure, organised in clusters of short branch chains, giving rise to a relatively compact macromolecular organisation within starch granules (Donald, Bertoft, Eliasson, & Hill, 2001; Pérez & Bertoft, 2010; Tester, Karkalas, & Qi, 2004; Vamadevan & Bertoft, 2015). Native starches contain 15–30 % amylose and 70–85 % amylopectin, with this ratio varying depending on individual cultivars, growth conditions and harvesting techniques. The properties of starch depend greatly on the molecular composition and structural organisation of its components, and have a significant impact on the material’s highly diverse applications (Morrison, Tester, Snape, Law, & Gidley, 1995; Tester et al., 2004).

Starch exhibits the ability to associate into a range of semi-crystalline forms, and form a variety of aggregated structures and hydrogels as a result of different processing conditions. Upon heating in excess water, native starch granules absorb water and swell. This is followed by the irreversible loss of granules’ molecular and crystalline order, amylose leaching into the surrounding medium, a process commonly referred to as starch gelatinisation (BeMiller, 2011). On cooling the dispersed composite glucans undergo lateral reassociation, forming cross-links and junction zones, the extent of which governs the short- and long-range order of the resulting three-dimensional network structure. With extended cooling and storage, this network is stabilised through further formation of inter- and intramolecular hydrogen bonds, a transition otherwise known as starch retrogradation (*i.e.* recrystallisation, †*ESI Figure S1*) (Biliaderis, 2009).

Previous studies have documented the complex behaviour exhibited by hydrothermally treated starch/water suspensions, pastes and gels, employing a range of rheological, thermal, diffraction, chromatographic and spectroscopic techniques (Colquhoun, Parker, Ring, Sun, & Tang, 1995; Evans & Haisman, 1980; Zobel, 2009). These works, along with ones featuring NMR spectroscopic characterisation of the ^1^H relaxation behaviour of the above systems (Kovrlija & Rondeau-Mouro, 2017; Ritota, Gianferri, Bucci, & Brosio, 2008; Vodovotz, Vittadini, & Sachleben, 2002), have led to the establishment of widely recognised and accepted models of the heterogenous arrangements within starch hydrogels.

As an easily accessible, renewable and environmentally friendly material, coupled with its broad range of rheological, physicochemical and biochemical properties, starch holds great promise for the development of “green” hydrogel-based materials (Biduski et al., 2018; Xiao & Yang, 2006; Xiao, 2013). It is important to recognise, however, that understanding the structuring, and inter-component interactions within starch hydrogels is paramount for the optimal tailoring of their physical and biochemical properties for directed applications. This is particularly challenging in the case of soft matter gels, due their structurally heterogenous nature, described by the simultaneous co-existence of both rigid and mobile components within a high water content assembly.

In this work we have applied multidisciplinary analytical techniques spanning a range of length scales, with an emphasis on solid-state and HR-MAS NMR spectroscopy, for probing structural heterogeneities within starch hydrogels. We report the existence of inherent mobile fractions within maize starch hydrogels, the magnitude of which appears to be negatively correlated with their amylose/amylopectin ratio and their rheological strength and structural rigidity. We have shown that the observed mobile moieties within the hydrogel matrix behave largely as highly solvated structures trapped within the porous hydrogel network, providing further support and new insight into the organisation and heterogenous structuring of starch hydrogels.

## 2 Materials and methods

### 2.1 Materials

Waxy maize, normal maize and amylomaize were purchased from Merck (formerly Sigma-Aldrich, Dermstadt, Germany). Hylon VII® and Hi-Maize 260® were kindly provided as a gift by Ingredion Incorporated (Manchester, UK). All other compounds and reagents were purchased from Merck.

### 2.2 Total moisture content

Moisture content (%) of all starch powders was measured using the weight loss following air-oven drying at 135 °C for 120 min, using the iAACC 44-19.01 official method (Eq. 1).(IAACC, 2009)

(1) $$\%\;Moisture\;Content=\;\frac{Initial\;Weight-Final\;Weight}{Initial\;Weight\;}\times 100$$

### 2.3 Total and apparent amylose content

This was performed as per the methodology of Knutson (Knutson, 1985) by dissolving the starch in an iodine solution (6.0 mM, dimethyl sulfoxide 70 % v/v in deionised water) overnight, followed by measuring the concentration of the amylose-iodine complexes in the solutions against a standard curve of amylose (from potato, >88 % pure, CAS 9005-82-7) using a UV–vis spectrophotometer (Biochrom Libra S50 UV/Vis Spectrophotometer, λ_max_ =600 nm).

The obtained apparent amylose content value was corrected to give the % total amylose content (*equation* 2).(C. A. Knutson, 1985)

(2) $$\%\;Amylose=\;\frac{\%\;Apparent\;Amylose-6.2}{93.8}$$

### 2.4 Swelling power

The swelling power of the five starch powder samples was analysed as per the methodology described by Leech et al. (Leach, McCowen, & Scoch, 1959). In brief, starch/water suspensions (0.1 % w/v) were incubated in a shaking water bath (60 °C, 150 rpm, 30 min), followed by centrifugation (1600 rpm, 20 min), removal of the supernatant and calculating the difference in mass (Eq. 3).

(3) $$Swelling\;Power=\;\frac{W_{wet}}{W_{dry}}$$

where *W_wet_* and *W_dry_* are the weights of the starch sample following centrifugation and removal of the supernatant, and the dry starch powder prior to the addition of water, respectively.

### 2.5 Hydrogel preparation

Gelatinisation and subsequent storage of all starch gel samples was performed in sets of three for each sample of interest by preparing 10 % (w/v) starch/deionised water suspensions in 25.0-mL Pyrex® screw-top vials, vortex mixed and autoclaved (121 °C, 15 psig) for 20 min, followed by their storage for the total duration of 8 days at three different temperature conditions, where one set was stored at a constant temperature of 4 °C for the whole duration of 8 days (*i.e.* low temperature isothermal), another one at 30 °C for the full duration of 8 days (high temperature isothermal), and the third set of starches was stored at 30 and 4 °C (4 consecutive days at each temperature, *i.e.* thermocycled conditions), all of which resulted in the formation of opaque white gels (†*ESI, Figure S2*).

Hydrogels intended for solution-state NMR experiments were prepared using D_2_O (99.9 % ^2^H) instead of H_2_O, following the exact same procedures as above, where the total starch/D_2_O suspension volume was 700 μL.

### 2.6 Differential scanning calorimetry (DSC)

DSC experiments were performed on a TA Instrument (TA Instruments Ltd., New Castle, USA) multicell differential scanning calorimeter (MC-DSC), equipped with three sample Hastelloy ampoules and one reference Hastelloy ampoule, where the reference pan was filled with deionised water. The furnace was continuously flushed with dry Nitrogen at a rate of 50 mL.min^−1^. For native starch samples, 100 mg of starch powder were made up with 0.9 mL degassed, deionised H_2_O (*i.e.* 10 % w/v suspensions), where the individual amount of water added to each sample was adjusted depending on their respective moisture content data. The vessels were hermetically sealed, and samples were analysed in the range of 10–150 °C, with a heating rate of 1 °C min^−1^, a cooling rate of 2 °C min^−1^, followed by a second heating cycle at 1 °C min^−1^. The slow heating rate was chosen in order to ensure that gelatinisation occurred under pseudo-equilibrium conditions (Bogracheva, Wang, Wang, & Hedley, 2002).

For all hydrogel samples, approximately 800 mg of stored sample were accurately weighed into each Hastelloy ampoule. The vessels were sealed, and all measurements taken against an empty reference ampoule. All gel samples were subjected to a single heating cycle at 1 °C min^−1^, followed by a single cooling step at 2 °C min^−1^. All thermal data was analysed using TA Universal Analysis software package, establishing the onset, peak and conclusion temperature (*T*_O_, *T_P_* and *T*_C_, respectively), and the overall enthalpy of each thermal transition (Δ*H*). All scans were run at least in triplicate and all obtained results are presented as means.

### 2.7 Sub-ambient DSC

Subambient DSC experiments were performed on a TA Instruments Discovery Series DSC2500, using TA Instruments Tzero® pans and hermetic lids (reference numbers 901683.901 and 901684.901, respectively), sealed using Tzero® sample press die. Approximately 3.5–5.0 mg of each hydrogel sample were loaded into each pan and hermetically sealed. All measurements were referenced against a sealed, empty pan. All gel samples were subjected to a single cooling cycle from 20 to -40 °C min^−1^, at 5 °C min^−1^, followed by a single heating step from -40 to 30 °C, at 2 °C min^−1^. The furnace was flushed with dry nitrogen at a rate of 50 mL.min^−1^. All thermal data was analysed using TA TRIOS software package, establishing the onset, conclusion temperature (*T*_O_, and *T*_C_, respectively), and the overall enthalpy of each thermal transition (Δ*H*). All scans were run at least in triplicate and all obtained results are presented as means.

### 2.8 Dynamic oscillatory rheology

Rheological analyses were performed on a TA Instrument (TA Instruments Ltd., New Castle, USA) Rheometer AR2000, with 13 mm parallel plate geometry, equipped with a Peltier device for temperature control, where hydrogel samples were carefully excised using a 13mm cork borer (Breckland Scientific Supplies Ltd., Stafford, UK) and cut into discs, 10 mm in height, using a surgical blade (Swann-Morton Ltd., Sheffield, UK). All samples were analysed at a constant temperature of 5.0 °C and the interplate gap was set to 10 mm. All starch hydrogels were loaded onto the sensor plate and allowed to equilibrate for 2 min to minimise the impact of loading effects and for temperature to equilibrate throughout the sample. Strain sweeps were performed in the range of 0.01–100 % at a constant frequency of 1.0 Hz to ascertain the materials’ linear viscoelastic (LVE) range. This was followed by frequency sweep analyses in the range of 0.1–10.0 Hz (*i.e.* 0.6283–62.83 rad/sec) at a constant strain of 0.3 % to determine the samples’ sinusoidal stress response with respect to the applied

strain (*γ*, Eq. 4).

(4) $$\gamma =\gamma_{0}\times \text{s}\text{i}\text{n}(\omega t+\;\delta )$$

where *γ*_0_ is the maximum amplitude of the response strain, ω is the oscillatory frequency (rad/s), *t* is the given time period, and *δ* is the phase angle shift of the sinusoidal stress with respect to the strain. This was followed by obtaining the samples’ storage and loss moduli (G’ and G’’, respectively, Eqs. 5 and 6) through the stress (σ_o_) and the strain components, where the ratio of these moduli is directly related to the samples’ *δ* values (Eq. 7) (Marangoni & Peyronel, 2014; Morrison, 2001).

(5) $$G^{'}=\sigma_{0}/\gamma_{0}\times \text{c}\text{o}\text{s}(\delta )$$

(6) $$G^{''}=\sigma_{0}/\gamma_{0}\times \text{s}\text{i}\text{n}(\delta )$$

(7) $$\text{tan}\left(\delta \right)=\;G''/G'$$

All measurements were taken at regular time intervals, analysed using TA Data Analysis software package, and presented as averages of a minimum of three runs, where deviation in the obtained parameters between runs of the same samples were less than 10 %.

### 2.9 Powder X-ray diffraction and estimation of long range order

Powder X-ray diffraction (PXRD) patterns of all starch powders were measured on an ARL™ X'Tra Powder Diffractometer (Thermo Scientific™). All samples were scanned with Cu *K_α_* radiation (λ =0.154 nm), and reflections were detected via a scintillation detector over the angular range of *2*θ = 5.0–54.99°, with a step interval of 0.01°, and step duration of 0.6 s. The X-ray generator was set at 45 kV and 40 mA. Approximately 600 mg of each sample were packed into the loading dish to a depth of 4 mm and levelled with a razor blade. Diffraction patterns of all starch hydrogels were obtained following freeze-drying of the gel samples and grinding using a mortar and pestle.

Longrange order of both powders and gels was estimated following the approach described by Gidley et al. (Lopez-Rubio, Flanagan, Gilbert, & Gidley, 2008). In brief, peaks in the diffraction patterns of all starch samples were manually fitted using a combination of Lorentzian and Gaussian functions, utilising the PeakFit (SigmaPlot, Systat Software Inc. ©) software package, where 10 peaks were selected for starches of A-type crystallinity (*i.e.* waxy maize and normal maize) and 11 peaks for starches of B-type crystallinity (*i.e.* amylomaize, Hylon VII® and Hi-Maize 260®), with the addition of an amorphous “background” peak in the range of *2*Θ 15−17° for each diffraction pattern (†*ESI, Figure S3*). The total percentile long-range order in our samples was calculated based on the ratio between the sum of the area under each crystalline peak and the total area under the whole diffractogram (Lopez-Rubio et al., 2008).

### 2.10 Solid-state NMR spectroscopy

#### 2.10.1 Cross Polarisation and Single Pulse Magic Angle Spinning (CP and CPSP/MAS) NMR Spectroscopy

Solid-state ^1^H-^13^C CP/MAS experiments were carried out for powder samples using a Bruker Avance III 300 MHz spectrometer, equipped with an HX 4-mm probe, at a ^13^C frequency of 75.47 MHz and MAS rate of 12 kHz. Approximately 100–120 mg of solid samples were packed into a 4-mm zirconium oxide rotor with a Kel-F end cap. The ^1^H-^13^C CP/MAS NMR experimental acquisition and processing parameters were π/2 ^1^H *rf* pulse length of 3.50 μs and π/2 ^13^C *rf* pulse length of 4.50 μs, a contact time of 1000 μs, a recycle delay of 10 s, and a minimum of 5120 number of scans. All ^1^H and ^13^C spectra were referenced with respect to tetramethylsilane (TMS). The measurements were carried out at approximately 25 °C.

Solid-state ^1^H-^13^C CP and CPSP/MAS NMR experiments were carried out for the starch gels using a Bruker Avance III 400 MHz spectrometer, equipped with an HXY 4-mm probe, at a ^13^C frequency of 100.64 MHz, and MAS rate of 6 kHz. Gel samples were packed into an insert, enclosed with a stopper and a screw cap, and placed inside a 4-mm cylindrical rotor with a Kel-F end cap. The ^1^H-^13^C CP/MAS NMR experimental acquisition and processing parameters were π/2 ^1^H rf pulse of 3.20 μs and π/2 ^13^C rf pulse of 3.86 μs, a contact time of 1000 μs, a recycle delay of 5 s, with a minimum of 6144 number of scans. ^1^H and ^13^C chemical shifts were referenced to tetramethylsilane (TMS). The spectra were measured at approximately 5 °C. These experiments were also performed at variable temperature (VT) in the range of +5.0 to -25.0 °C in steps of 5 °C, where these were performed with a total of 2000 number of scans per temperature step and all other parameters were kept the same.

Shortrange starch molecular ordering was estimated using the method described by Flanagan et al. (Flanagan, Gidley, & Warren, 2015). In brief, following the acquisition of the free induction decay, the data were Fourier transformed, phase corrected and zero-filled to 4096 data points. The spectra were then subjected to partial least squares fit using a large library of experimental ^1^H-^13^C CP/MAS NMR spectra of both raw granular and processed starches of various botanical origins, featuring all crystalline polymorphs (A-, B- and V-type). The short range order of the samples was obtained from the fit.

#### 2.10.2 Spectral deconvolution

Spectral deconvolution was performed using the MestReNova software v14 (MestreLab Research©) at high resolution with a minimum of 20 fitting cycles, using a mixture of Lorentzian and Gaussian functions, with minimal manual adjustment of peak position. This was performed iteratively until the acquisition of minimal outlier residuals.

#### 2.10.3 Estimation of mobility

Estimation of mobility levels across all peaks of interest was calculated as shown below (Eq. 8).

(8) $$\%\;Mobility=\frac{I_{CPSP}-I_{CP}}{I_{CPSP}}\times 100$$

where *I_CPSP_* and *I_CP_* are the ^13^C peaks’ normalised intensity values in their CPSP and CP/MAS NMR spectra, respectively.

#### 2.10.4 ^13^C Direct Polarisation (DP) with High Power ^1^H Decoupling (HPDEC) NMR Spectroscopy

DP experiments were performed on a Bruker AVANCE III 850 MHz solid-state NMR spectrometer (UK National 850 Solid-State NMR facility at the University of Warwick) equipped with a 4 mm HX H13892B probe, using 90° angle on ^13^C of 3.5 μs, relaxation delay of 2 s, at MAS rate 10 kHz, at 5 °C and with a minimum of 256 scans.

Further DP experiments with long relaxation delays were performed on the above Bruker Avance III 400 MHz spectrometer, equipped with an HXY 4-mm probe. The acquisition parameters were 90° angle on ^13^C of 3.3 μs, recycle delay of 10, 20 and 150 s, at MAS rate of 6 kHz, at 5 °C, and with a minimum of 1024 scans.

### 2.11 HR-MAS NMR spectroscopy

Direct ^13^C detection and ^1^H-^13^C HSQC experiments on starch gels were carried out on a Bruker Avance III 800 MHz spectrometer, equipped with a high-resolution magic angle spinning (HR-MAS) 4 mm double resonance probe. All experiments were carried out at 5 °C and at MAS rate of 6 kHz.

The ^13^C direct detection experiments were carried out with a 90° ^13^C pulse of 6.7 μs, a relaxation delay of 1.0 s and a minimum of 4k scans, and the 2D ^1^H-^13^C HSQC experiments were performed with a π/2 flip angle of 7.72 μs, relaxation delay of 5.0 s and 128 increments in the indirect (F1) dimension.

^1^H longitudinal relaxation times (*T*_1_) were measured using the inversion recovery pulse sequence, using recycle delay of 10 s. A total of 16 points were recorded with time delays ranging from 0.05 to 20 s for all hydrogel samples. This was also performed at variable temperature in the range of +5.0 to -15.0 °C in steps of 5 °C for normal maize starch hydrogels. The evolution of spectral intensities of all ^1^H peaks of the starch hydrogels and the one for HDO were mathematically fitted to the monoexponential function below (Eq. 9):

(9) $$M_{z}\left(\tau \right)=M_{0}\times [1-2e^{\left(\frac{-\tau }{T_{1}}\right)}]$$

where *M_z_* is the z-component of magnetisation, *M_0_* is the equilibrium magnetisation and *τ* is the time delay.(Claridge, 2016; Ramalhete et al., 2017)

### 2.12 Solution-state NMR spectroscopy

^13^C Solution-state NMR spectra were acquired on a Bruker Avance I spectrometer, operating at ^13^C frequency of 125.79 MHz, equipped with a 5 mm probe. Hydrogels were prepared directly in Pyrex® NMR tubes (Norell Inc.®), starting with 10 % (w/v) starch/D_2_O suspensions with total volume of 700 μL and following all other gelatinisation and storage procedures as described above (*see Hydrogel preparation section*). All ^1^H and direct ^13^C detection experiments were acquired with a 10 μs ^13^C *rf* pulse, 2.0 s relaxation delay, a minimum of 2000scans and carried out at 25 °C. The short recycle delay was chosen to probe the structure of the liquid-like components in the hydrogels.

### 2.13 Water Polarisation Transfer Cross Polarisation (WPT-CP) NMR Spectroscopy

WPT-CP NMR experiments have been shown to be particularly useful for probing waterpolysaccharide interactions in semisolid materials, for instance plant cell walls (White, Wang, Park, Cosgrove, & Hong, 2014). The experiment starts by filtering out ^1^H magnetisation from immobile components (*e.g*. starch particle network; short *T*_2_) while keeping ^1^H magnetisation from mobile components (*e.g*. water; long *T*_2_). Subsequently, the latter is transferred to the particle network during a variable mixing period (*t*_mix_) via three different mechanisms, (i) ^1^H spin diffusion, (ii) ^1^H intermolecular nuclear Overhauser effect (nOe) and (iii) ^1^H chemical exchange (White et al., 2014). It should be noted that, in comparison with mechanisms (i) and (iii), nOe contributes to a lesser extent to magnetisation transfer at low-tomoderate MAS rates (White et al., 2014). Finally, ^1^H magnetisation is transferred to ^13^C via CP and the ^13^C spectrum is acquired under conditions of high power ^1^H decoupling (†*ESI, Figure S4*).

The acquisition parameters were 90° ^1^H and ^13^C rf pulses of 3.20 and 3.86 μs, respectively. A total *T_2_* filter period (2Δ + 180° pulse length) of 4 ms and mixing times of 25 ms was used. To minimise the contribution of intramolecular spin diffusion during the CP period a contact time of 500 μs was employed. The short mixing time and low CP efficiency of starch hydrogels precluded running complete WPT-CP build-up curves at variable mixing times. All WPTCP experiments were carried out at 5 °C, MAS rate of 6 kHz with a minimum of 6k scans. Peak intensities were normalised against a reference spectrum obtained with very short *T_2_* filter duration and mixing times (both set as 1.0 μs). The percentage of water polarisation transfer (% WPT-CP) was calculated for C-1, C-2,3,5 and C-6 carbon peaks as the ratio of the peak intensities at 25 ms mixing time (*i.e.* build-up) and the reference spectrum (Eq. 10)

(10) $$\%\;WPT-CP=\frac{A_{25ms}}{A_{REF}}\times 100$$

where A_25ms_ and A_REF_ are the carbon peak intensities of WPT-CP spectra acquired at 25 and 0 ms, respectively. The C-4 peak was disregarded, due to its low intensity. The peak intensity in WPT-CP curves depends on the number, distance and mobility of water molecules at a given ^13^C environment. Hence, peaks corresponding to less sterically constrained vicinities showing faster WPT-CP growth at shorter mixing times (*i.e.* 25 ms), compared to sterically hindered ones.

### 2.14 Statistical analyses

A two-tailed, paired *t*-test was performed on all quantitative NMR measurements with a confidence interval of 95 %. All statistics results can be seen in tables S8-S13 in the ESI.

## 3 Results and discussion

In this work we selected of group of maize starches, which we used for the hydrothermally preparation of hydrogels with a range amylose and amylopectin content. We used these as model systems to derive structure-function relationships, linking bulk structural and motional features to properties and function. Previous works have shown that different conditions and methods of starch gelatinisation and retrogradation can induce changes in the materials’ macroscopic properties. In this work we aimed to probe the structural origin of these observations for the development of new functional materials.

### 3.1 How does starch structure and composition determine gelling and macroscopic gel properties?

We characterised the composition and properties of the granular starches used for the production of hydrogels. The results of moisture and amylose contents, swelling power and short- and long-range orders of all five starch powders are presented in Table 1. All results are in good overall agreement with previously reported data (Bertoft, 2017; Lopez-Rubio et al., 2008; Patindol, Gu, & Wang, 2009; Pérez & Bertoft, 2010; Tester & Morrison, 1990; Us, Gonenc, Kibar, & Aytunga, 2010).

**Table 1:** Material characterisation data of all five maize starch powder samples.

Starch Type	Moisture Content (%)	Amylose Content (%)	Swelling Power (wet/dry weight)	Short-Range Order via CP/MAS NMR (%)*	Long-Range Order via PXRD (%)**
Waxy Maize	12.2 (±0.2)	1.1 (±0.6)	10.7 (±0.1)	38.4 (±1.5)	33.7 (R^2^ = 0.9800)
Normal Maize	12.2 (±0.2)	23.1 (±0.4)	13.3 (±0.9)	36.6 (±1.5)	30.3 (R^2^ = 0.9900)
Amylomaize	11.3 (±0.1)	51.8 (±0.5)	4.6 (±0.3)	25.3 (±1.0)	23.0 (R^2^ = 0.9810)
Hylon VII®	11.5 (±0.6)	69.5 (±0.4)	4.8 (±0.5)	21.8 (±0.9)	19.2 (R^2^ = 0.9800)
Hi-Maize 260®	11.2 (±0.2)	69.7 (±0.7)	2.8 (±0.2)	30.8 (±1.2)	29.2 (R^2^ = 0.9910)

* Flanagan et al. (2015).

** Lopez-Rubio et al. (2008).

### 3.2 Bulk properties of starch hydrogels

#### 3.2.1 Thermal properties

**Fig. 1:**
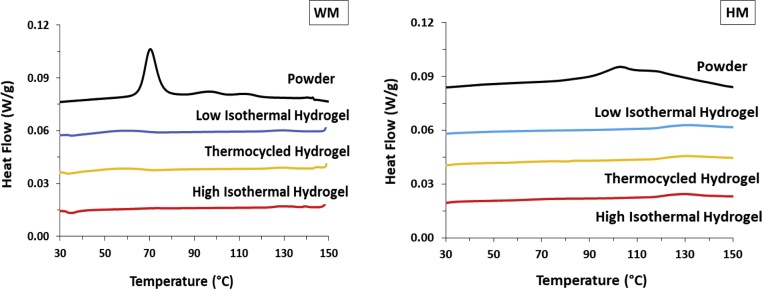
Composite DSC thermograms of waxy maize (WM) and Hi-Maize 260® (HM) starch powders, and their corresponding hydrogels prepared under different storage conditions. Curves have been offset for visual clarity purposes.

Example DSC thermograms of all waxy maize and Hi-Maize 260® starch powders and hydrogels can be seen in Fig. 1. The data for all other samples are presented in Figure S5 (*†ESI*), along with the thermal parameters defining each endothermic transition detected in their respective thermograms in Tables S1-4 (†*ESI*), where the data collected for the starch powder samples were in good overall agreement with previously published data (Liu, Yu, Chen, & Li, 2007; Russell, 1987).

Up to four distinct endothermic transitions were identified from the DSC data, where all thermal parameters are in agreement with previous works (Donovan, 1979; Liu, Yu, Simon, Dean, & Chen, 2009; Lopez-Rubio et al., 2008; Russell, 1987; Waigh, Gidley, Komanshek, & Donald, 2000; White, Abbas, & Johnson, 1989; Wronkowska, Soral-Smietana, Komorowska-Czepirska, & Lewandowicz, 2004). In the case of amylomaize, Hylon VII® and Hi-Maize 260®, all endothermic transitions occurred as one broad multitherm lacking clearly differentiated maxima (*i.e. T_P_*), which could be linked to a broader distribution of crystalline structural arrangements, compared to the ones in waxy and normal maize (Waigh et al., 2000; Zhou, Hoover, & Liu, 2004). The onset of gelatinisation of Hi-Maize 260® was delayed significantly compared to amylomaize and Hylon VII®, indicative of the greater crystallite stability and greater level of long-range ordering in the sample, further supported by its higher degree of crystallinity and short-range ordering, as per our PXRD and ^13^C CP/MAS NMR data, respectively. The majority of thermal transitions detected in the starch powders were of significantly lower enthalpy in their hydrogel analogues (J/g of the carbohydrate component), where the transitions associated with amylopectin crystallites were almost entirely absent (*†ESI, Table S3-5*). Furthermore, low isothermal and thermocycled storage conditions resulted in marginally greater helical reassociation, as evidenced by the slightly higher enthalpy of transitions compared to their high isothermal hydrogel counterparts. These observations are in agreement with previous works (Park, Baik, & Lim, 2009), and confirm the lower degree of short-range ordering in the hydrogel systems and further support the notion that retrogradation gives rise to molecular packing with distinct features, dissimilar to the ones observed in their powder analogues (*i.e.* prior to hydrothermal treatment, †*ESI*, Fig. 1).

#### 3.2.2 Mechanical properties

**Fig. 2:**
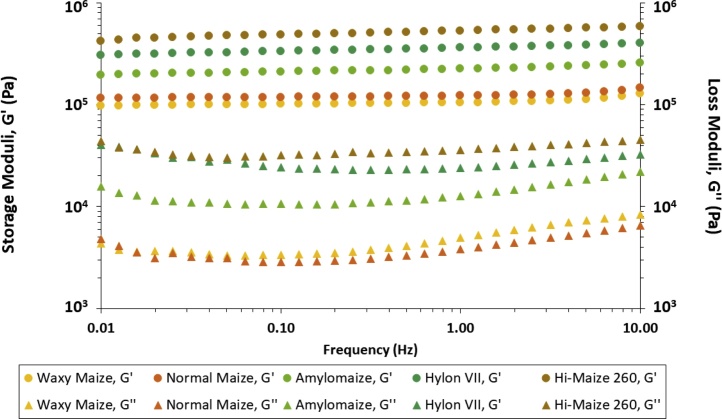
Storage and loss moduli (G’ and G’’, respectively) of all five maize starch hydrogels stored at low temperature isothermal conditions, as a function of applied frequency. Results presented are averages of a minimum of three repeats.

**Fig. 3:**
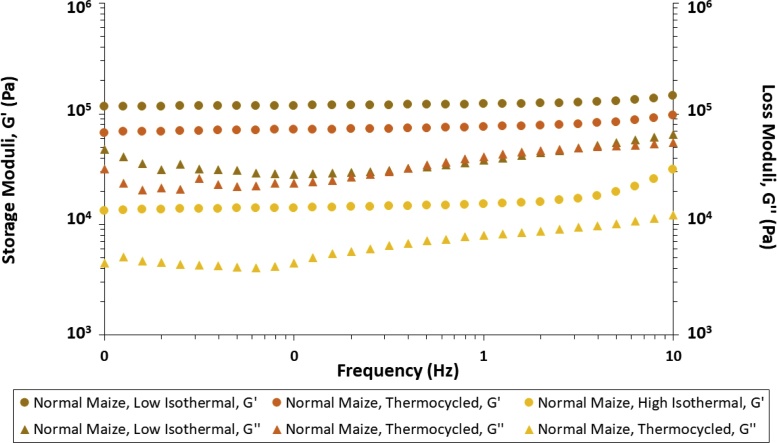
Storage and loss moduli (G’ and G’’, respectively) of all normal maize starch hydrogels stored at all three different conditions. Results presented are averages of a minimum of three repeats.

The results from the rheological investigations can be found in Fig. 2, Fig. 3, and S6 (†*ESI*). measurements. All hydrogels’ properties were in agreement with the established classic gel network parameters, documented in the works Burchard and Ross-Murphy (Burchard & Ross-Murphy, 1990) and Almdal et al. (Almdal, Dyre, Hvidt, & Kramer, 1993).

The extent of the LVE range of the hydrogels spanned from 0.03 to 0.5 % strain (†*ESI, Figure S6*). At low strain (*γ* = 0.3 %) the dynamic storage moduli (G’) remained independent of changes in the applied frequency until 1.0 Hz across all hydrogel samples, while their loss moduli (G’’) exhibited marginal fluctuations, typical of hydrogel-type materials (Van Den Bulcke et al., 2000), potentially indicating low levels of compensatory structural rearrangements of glucan chains and/or redistribution of water fractions within the hydrogel network in its dissipation of stress throughout the system.

The viscoelastic solid nature of our starch hydrogels was confirmed by their low *tan*(*δ*) values, ranging from 0.024 to 0.152, characteristic of strong soft matter materials (Harrats, 2009). Upon analyses of the rheological profiles of all maize starch hydrogels, there was a correlation (R^2^ = 0.9305, *Figure S7,* †*ESI*) between amylose content and storage modulus values (G’). This was in agreement with previous works (Xie et al., 2009; Xie, Halley, & Avérous, 2012). Since G’ values are related to the rigidity (*i.e.* density of junctions and cross-linking) of a viscoelastic material (Khan, Royer, & Raghavan, 1997), the dependence of the storage moduli of starch hydrogels on their individual concentration and total amylose and amylopectin content has been interpreted to be a consequence of the phase transformations occurring during the biphasic model of retrogradation, resulting in the formation of hydrogels – namely the inter-glucan chain reassociations and formation of junction zones (Lewen et al., 2003). Following granular decomposition and amylose leaching into the surrounding medium (†*ESI, Figure S1*), the greater kinetic energy in the system facilitates the inter-glucan entanglement, the susceptibility to which is dependent on the amylose and amylopectin macromolecular structural parameters, where the predominantly linear amylose chains can partially align and interact with each other with far greater ease on the timescale of our experiments (8 days total storage duration), compared to the globular/ellipsoid highly branched amylopectin clusters, due to the lower steric effects experienced by the former when compared to the latter of the two glucans (Hoover, 1995). It is also worth noting that unlike the consistent and progressive increase in storage moduli with increasing amylose content observed for the majority of the samples, upon inspection of all starch hydrogels’ individual G’’ values, it was evident that it was normal maize starch hydrogels that exhibited the lowest loss moduli, and not waxy maize starch (approximately 2.99 kPa *vs* 3.63 kPa, at 0.25 Hz, respectively), pointing towards the presence of multiple contributions to the hydrogels’ rheological properties, exceeding their individual amylose and amylopectin content. Overall, our results demonstrate that the synthesised maize starch hydrogels exhibit significant rheological strength, with G’ values comparable to, and in some cases higher than, synthetic and natural-synthetic hybrid gels, commonly employed in the pharmaceutical industry (Amiri et al., 2018; Shalviri, Liu, Abdekhodaie, & Wu, 2010).

### 3.3 Internal structural organisation, dynamics and inter-component interactions

#### 3.3.1 Long-range ordering

XRD patterns of all starch powders and corresponding low temperature isothermally stored hydrogels (†*ESI, Figure S8*) were in good overall agreement with previous data where available (Lopez-Rubio et al., 2008). On hydrothermal treatment and storage, the low-amylose containing starches produced gels with practically no detectable long-rage ordering. The only discernible features were observed as broad reflections in normal maize gels, indicative of the presence of V-type amylose crystallites (Liu, Xie, & Shi, 2016; Lopez-Rubio et al., 2008), and a change in helical packing from A- to the more stable B-type allomorph (†*ESI, Figure S9*) (Bulpin & Gidley, 1987; Waigh et al., 2000).

In contrast, the high-amylose samples yielded hydrogels of significantly more pronounced B-type organisation, and higher degree of ordering, further confirming their decreased susceptibility to hydrothermal treatment, evidenced by their delayed thermal transitions (†*ESI, Figure S5*). These observations were most likely also influenced by the differences in the retrogradation rates between amylose and amylopectin (hours *vs* days, respectively) (Jayakody & Hoover, 2008; Raigond, Ezekiel, & Raigond, 2015; Tester, Debon, & Sommerville, 2000).

#### 3.3.2 Short-range order and dynamics by solution-, gel- and solid-state NMR spectroscopy

Cross-polarisation (CP) allows for the observation of local structure and organisation, depending on the efficiency of polarisation transfer from ^1^H to ^13^C nuclei. Broad carbon peaks in ^1^H-^13^C CP/MAS NMR spectra of starch typically correspond to disordered regions, while sharp lines are indicative of carbons in ordered arrangements (Tang & Hills, 2003).

Assignment of ^13^C chemical shifts in all NMR spectra (†*ESI*, *Table S5*) was performed based on previous works and is shown in all NMR spectra (Gidley & Bociek, 1985; Gidley et al., 1995; Tan, Flanagan, Halley, Whittaker, & Gidley, 2007; Tang & Hills, 2003). The ^1^H-^13^C CP/MAS NMR spectra of maize starch powders and their corresponding low temperature isothermally stored hydrogels (†*ESI, Figures S10 and S11*) show considerably increased resolution in the hydrogel spectra. Hydration and hydrothermal treatments have been shown to increase mobility in the more structurally disordered fractions of starch (Baik, Dickinson, & Chinachoti, 2003; Tang & Hills, 2003). This has been linked to the state of the amorphous component in starches, which is highly susceptible to hydration effects, depending on whether it is in the glassy or rubbery state, respectively (*see sub-ambient NMR and DSC data below,* †*ESI Figures S16 and S17, and Table S6*) (Bogracheva, Wang, & Hedley, 2001).

The ^1^H-^13^C CP/MAS NMR spectra of all five maize starch hydrogels revealed differences primarily in the shoulder region of 100–104 ppm (green dashed line, †*ESI Figure S12*), ascribed to a combination of amorphous and V-type amylose crystalline arrangements (Veregin, Fyfe, & Marchessault, 1987). These differences were most pronounced when comparing low to high amylose starch hydrogels (*e.g.* waxy maize to Hi-Maize 260® gels, †*ESI Figure S12*). Waxy maize gels displayed a broader chemical shift distribution compared to Hi-maize 260® gels, which was expected, as a consequence of the latter’s higher V-type amylose content, as evident by its PXRD diffraction patterns (†*ESI, Figures S3 and S8*) (Baik et al., 2003; Tan et al., 2007; Zhang, Dhital, Flanagan, & Gidley, 2014).

**Fig. 4:**
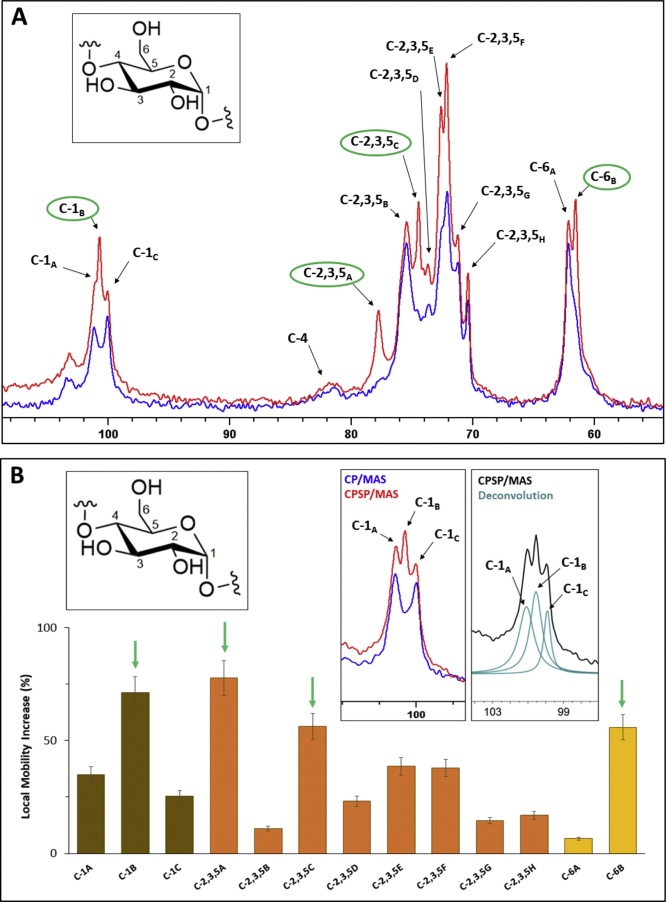
(A) An overlay of CP and CPSP/MAS NMR spectra of low temperature isothermally stored normal maize hydrogels with peak assignment. Peaks of significantly increased local mobility are shown in green. (B) Bar chart of estimated levels of local mobility per signal across all carbon nuclear environments with arrows indicating peaks of increased local mobility. Inlays showing spectral excerpts featuring composite peak deconvolution and assignments (For interpretation of the references to colour in this figure legend, the reader is referred to the web version of this article.).

Comparison of ^1^H-^13^C CP and CPSP/MAS NMR spectra for starch hydrogels revealed the presence of previously undocumented structural domains of significant local mobility existing in C-1, C-2,3,5 and C-6 environments, in all maize variety hydrogels (Fig. 4 *and* †*ESI, Figure S13*). Several of these peaks are only observable under CPSP conditions (Fig. 4A*, in green*), indicative of their considerable mobile behaviour, rendering them undetectable under CP conditions. Spectral deconvolution revealed the presence of three separate peaks within the anomeric carbon (C-1) environment (99.0–101.8 ppm), where the side peaks (99.8 and 100.8 ppm) exhibited significantly lower local mobility levels, compared to the central peak (100.3 ppm) within the C-1 environment (Fig. 4A and B). All three peaks were present in the spectra of all hydrogels. These data further confirm the changes in helical packing in our hydrogels compared to their powder analogues, initially observed in our PXRD data (*see above*), and also supported by PXRD analyses in previous works (Baik et al., 2003; Gidley & Bociek, 1985; Kalichevsky, Orford, & Ring, 1990). Full spectral deconvolution (†*ESI, Figure S14*) allowed for the quantification of local mobility at each ^13^C site (*see Materials and Methods section,* Eq. 6).

**Fig. 5:**
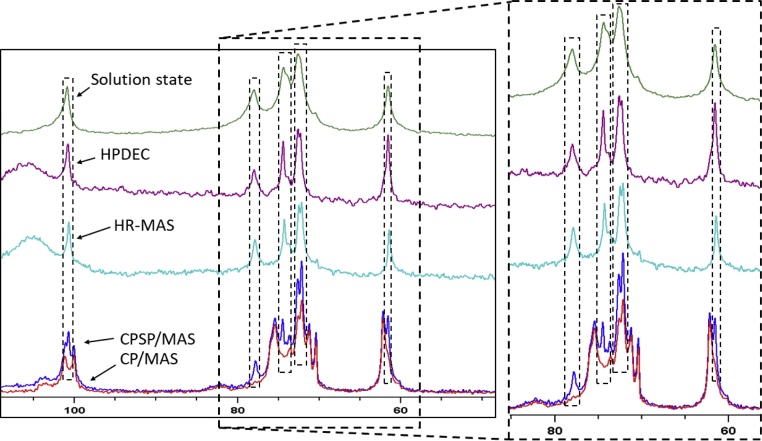
Overlay of ^13^C{^1^H} HR-MAS (turquoise), ^13^C{^1^H} solution state (green), ^13^C{^1^H} MAS (recycle delay of 2 s, purple) and ^1^H-^13^C CP and CPSP/MAS solid-state NMR spectra (red and blue, respectively) of low temperature isothermally stored waxy maize starch hydrogel in D_2_O. Dashed lines shows overlapping peaks. A zoomed-in image of the C-2,3,5 and C-6 chemical shift region is shown in inlay (For interpretation of the references to colour in this figure legend, the reader is referred to the web version of this article.).

**Fig. 6:**
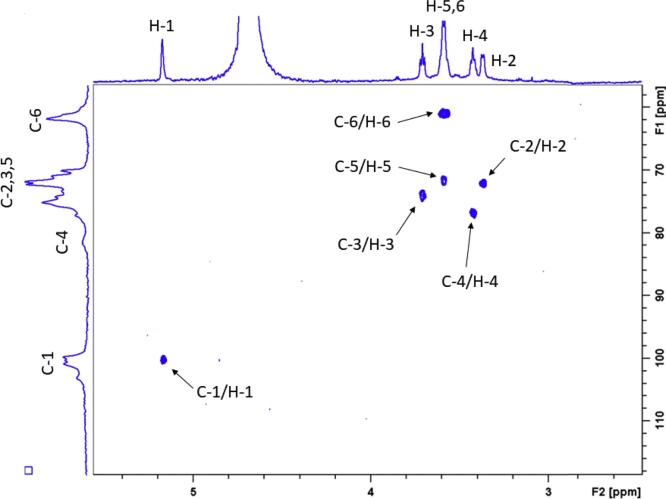
^1^H-^13^C HSQC NMR spectrum of low temperature isothermally stored amylomaize starch hydrogel with peak and cross-peak assignment. The ^13^C spectrum in the indirect (F1) dimension is a ^1^H-^13^C CPSP/MAS NMR spectrum.

Combined application of solid-, solution-state and HR-MAS ^13^C NMR experiments revealed that the peaks observed in solution-state and HR-MAS spectra correspond to those detected through ^1^H-^13^C CPSP/MAS NMR experiments (Fig. 5). The rigid components in the starch hydrogels became observable under longer recycle delay (150 s) conditions (*†ESI Figure S15*). This led to the hypothesis on the origin of these peaks being largely solvent-exposed, structurally mobile starch fractions within the hydrogel network. The observed line broadenings across all peaks in our solution state spectra was expected due to the solid-like state of the overall starch hydrogel matrix and the anisotropy of the chemical shifts. Chemical shift anisotropy (*i.e.* orientation dependence) results from the non-spherically symmetrical (and hence orientation dependent) electron density distribution around nuclei, and its orientation along the external magnetic field. These were partially mitigated under HR-MAS conditions, resulting in improved signal resolution, evident by the lower line broadening compared to solution-state experimental settings (Fig. 5). These conclusions were further supported by the observed cross-peaks in the ^1^H-^13^C HSQC NMR spectra (Fig. 6 *and †ESI Figure S16*), *i.e*. the ^1^H peaks give cross-peaks only with the ^13^C peaks corresponding to the mobile component.

The population of mobile components decreased progressively with lowering of the temperature. The peaks corresponding to mobile species are not detectable in the range of 10.0 to -25.0 °C (†*ESI Figures S17 and S18*). This was in agreement with the hydrogels’ longitudinal relaxation times (*T_1_*) across all ^1^H nuclei, which became significantly lower at temperatures below -10.0 °C (†*ESI, Figure S19*), confirming the transition from gel- to solid-like state of the hydrogel matrix.

DSC data confirmed that the majority of the water present in our starch hydrogels behaves as freezeable bound water, where the exothermic freezing event of this population occurred in the range of 13.2 to -23.5 °C (†*ESI, Table S6*). This was in agreement with the relative longitudinal relaxation (*T_1_*) times of the water molecules (*i.e.* the HDO peak) in the starch hydrogels, where *T_1_* values of the water fraction in the hydrogels were considerably lower than the expected *T_1_* for free water molecules (†*ESI, Table S7*), indicative of restricted molecular motions.

The findings from the combined application of these advanced NMR techniques led to our hypothesis on the origin of these mobile fractions being highly solvent-exposed and/or partially solvated starch matrix structural elements trapped within the overall rigid hydrogel macroscopic matrix, the physicochemical properties of which are being dominated by the surrounding water environment. Together these structurally and motionally distinct moieties comprise the overall structural heterogeneity of starch hydrogel systems. These data build on earlier works on low-temperature NMR spectroscopic experiments on starches (Colquhoun et al., 1995; Larsen, Blennow, & Engelsen, 2008).

#### 3.3.3 Role of water in hydrogel organisation and integrity

**Fig. 7:**
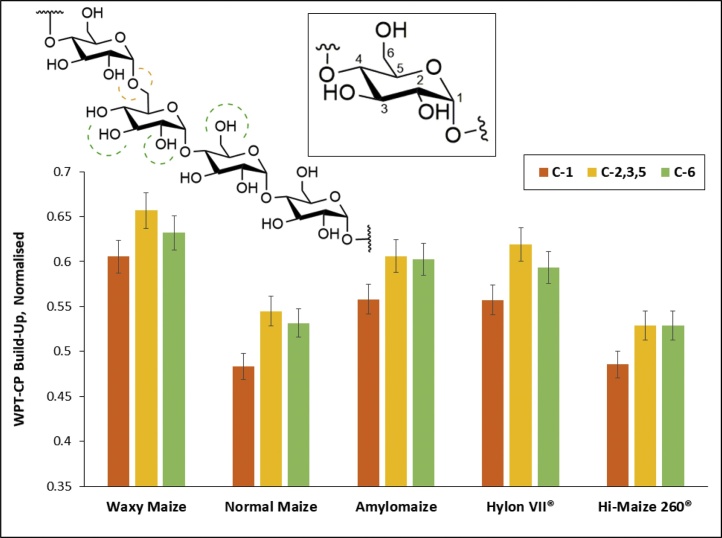
WPT-CP build-up across all five low temperature isothermally stored maize hydrogels (t_mix_ =25 ms). Inlays showing a glucose monomer and part of a starch chain featuring [1→4] and [1→6]-α linkages, where dashed lines indicate the relative steric constraints around nuclei, with green indicating low, orange – moderate and red – high levels of steric confinement. Error bars based on the S/N ratio of our spectral data (For interpretation of the references to colour in this figure legend, the reader is referred to the web version of this article.).

Comparative WPT-CP build-up graphs indicating the differences in rate of polarisation transfer from water molecules within our hydrogels to individual ^13^C nuclei of the starch matrix can be seen in the ESI (*†ESI, Figure S20*). Comparison of the levels (normalised) of transferred magnetisation at the point of build-up (*t*_mix_ =25 ms) revealed that water molecules are more structured around C-2,3,5 and C-6 nuclei, compared to C-1 (Fig. 7 *and †ESI, Figure S20*). This was explained by the latter’s involvement in [1→4]-α bonds within starch, contributing to a more confined vicinity around these nuclei (red dashed line, Fig. 7), compared to C-6, due to its lesser involvement in glycosidic linkages (*i.e.* [1→6]-α bonds making up the branching points within amylose and amylopectin). These observations were consistent across all our hydrogel samples. The C-4 peak was disregarded in this analysis, due to its low intensity (*see Materials and Methods section*). It should be noted that *t*_mix_ =25 ms was used for comparison between data sets, as it represented a point during the signal build-up phase (*i.e.* rate of polarisation transfer), whereas almost all of the signal was recovered at *t*_mix_ =100 ms across all samples, indicating nearly complete transfer of initial magnetisation.

Comparing the WPT-CP data per individual nuclei across all our hydrogels at the point of magnetisation build-up (*i.e. t*_mix_ =25 ms, †*ESI Figures S20*), no consistent significant differences were observed between our samples. The five maize starch hydrogels behaved in a very similar manner with respect to their interactions with structured proximal water molecules. This was also supported by saturation transfer difference (STD) NMR experiments (*data not shown*) probing the rate of transfer of magnetisation through space (by nOe) from the bulk hydrogel backbone to the water molecules within the hydrogel network. This offers further support for the origin of the observed mobile starch moieties, as it eliminates the organisation of water molecules within the hydrogel network being the governing factor for these mobile starch fractions.

#### 3.3.4 Effect of storage conditions on local mobility

**Fig. 8:**
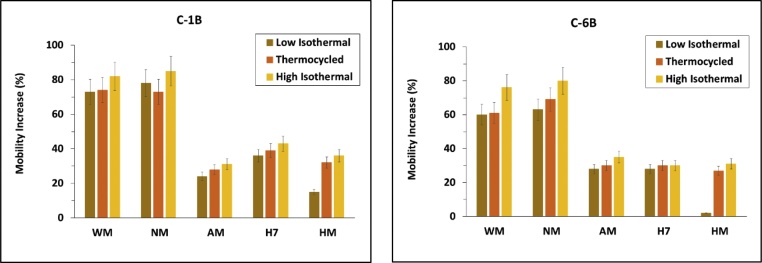
Overall increase in local structural mobility in waxy maize (WM), normal maize (NM), amylomaize (AM), Hylon VII® (H7) and Hi-Maize 260® (HM) starch hydrogels, organised by ^13^C nuclear environment with C-1B (left), and C-6B (right).

The levels of local mobility varied according to hydrogel storage conditions, in the series high isothermal > thermocycled > low isothermal hydrogels. Furthermore, hydrogel local structural mobility correlated with their levels of rheological resilience (R^2^ = 0.8511, †*ESI, Figure S21)*, where low-amylose and structurally weaker gels exhibited higher local mobility levels (Fig. 8) and *vice versa* (†*ESI, Figure S22*).

This was interpreted as being a consequence of the differences in reassociation and retrogradation of amylose and amylopectin, where the faster reassociation rate of amylose gave rise to a more tightly organised network, restricting the motional freedom of the solvated starch chains. Previous works have shown that retrogradation storage temperature and amylose content have a significant impact on the rheological properties of cooked starches (Park et al., 2009; Xie et al., 2009). In this work, we provide further insight into the mechanisms behind these observations.

## 4 Conclusions

We aimed to gain more information about the structural arrangements within starch hydrogels governing their physicochemical properties, with the purpose of developing functional future materials for advanced structural, cosmetic, and pharmaceutical applications.

All ^13^C sites were found to exhibit increased local structural mobility in their ^1^H-^13^C CPSP/MAS NMR spectra, which were conserved across all maize starch hydrogels explored in this study. These newly documented structural elements of increased local mobility behaved largely as solvated chains in the water phase of our hydrogel matrices, and correlated with other physicochemical and macromolecular properties, such as individual glucan composition and rheological strength. Our findings offer new insight into previously unreported structural features of starch hydrogels, which appear to be related to their macroscopic and biochemical properties, thus facilitating the tailored study and design of novel, environmentally friendly structural soft matter materials for future use. Further experimental procedures are necessary in order to ascertain the full impact of these mobile structural moieties in starch hydrogels on their extended physicochemical and biochemical properties.

## CRediT authorship contribution statement

**Todor T. Koev:** Investigation, Formal analysis, Writing - original draft, Visualization. **Juan C. Muñoz-García:** Investigation, Methodology, Writing - review & editing. **Dinu Iuga:** Resources, Methodology. **Yaroslav Z. Khimyak:** Conceptualization, Supervision, Funding acquisition, Writing - review & editing. **Frederick J. Warren:** Conceptualization, Supervision, Funding acquisition, Writing - review & editing.

## Declaration of Competing Interest

The authors declare no competing financial interest.
