# Supplementary material for: Structural heterogeneities in starch hydrogels
Source: Carbohydr Polym. 2020 Dec 1;249:116834. doi: 10.1016/j.carbpol.2020.116834 (PMC7519636; doi:10.1016/j.carbpol.2020.116834)
Supplement: Supplementary file 1 [file mmc1.docx]

**Structural Heterogeneities in Starch Hydrogels**
*Electronic Supplementary Information*

Todor T. Koev ^1,2^, Juan C. Muñoz-García ^1^, Dinu Iuga ^3^, Yaroslav Z. Khimyak ^1^, Frederick J. Warren ^2^

*1 School of Pharmacy, University of East Anglia, Norwich Research Park, NR4 7TJ (UK)*

*2 Food Innovation and Health, Quadram Institute Bioscience, Norwich Research Park, NR4 7UQ (UK)*

*3 Department of Physics, University of Warwick, Coventry CV4 7AL (UK)*

**List of Contents**

1. Diagrams and Photos of Starch Hydrogels
2. Differential Scanning Calorimetry (DSC)
3. Rheology
4. Powder X-ray Diffraction (PXRD)
5. Nuclear Magnetic Resonance (NMR) Spectroscopy
6. Statistical Analyses
7. **Diagrams and Photos of Starch Hydrogels**


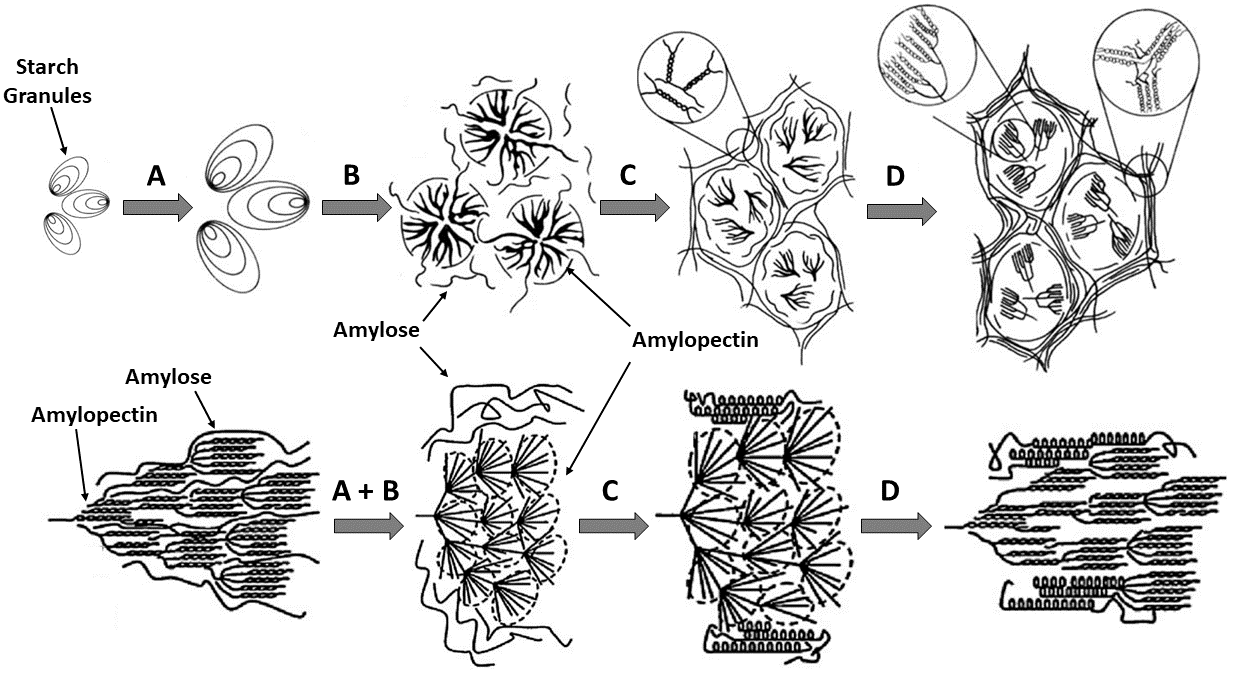


*Figure S1: Graphical representation of starch gelatinisation and retrogradation, indicating each individual stage: granule swelling (A), granule disruption and amylose leaching (B), glucan reassociation on cooling (C) and further glucan association and cross-linking with storage (D), adapted from the works of Goesart et al.* (Goesaert et al., 2005) *and Yu and Christie* (Yu & Christie, 2005).


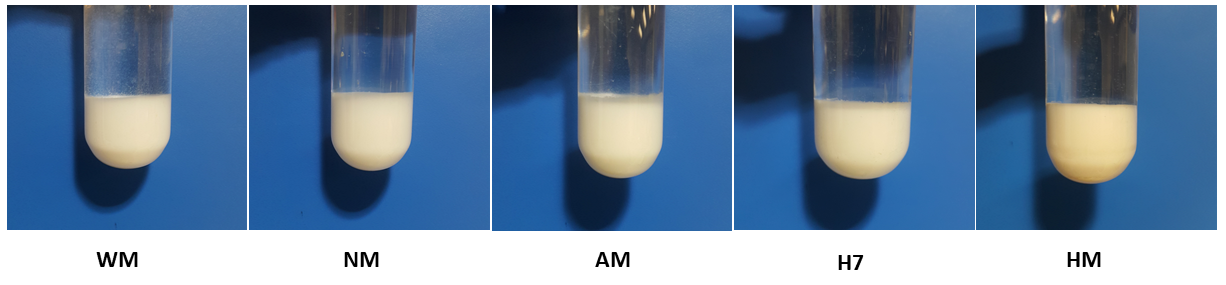


*Figure S2: Photo of end of storage duration (day 8) of waxy maize (WM), normal maize (NM), amylomaize (AM), Hylon VII™ (H7) and Hi-Maize 260™ (HM) hydrogels, in their preparation vessels (25-mL Pyrex® vials), prepared as per Materials & Methods section.*

1. **Differential Scanning Calorimetry (DSC)**

| 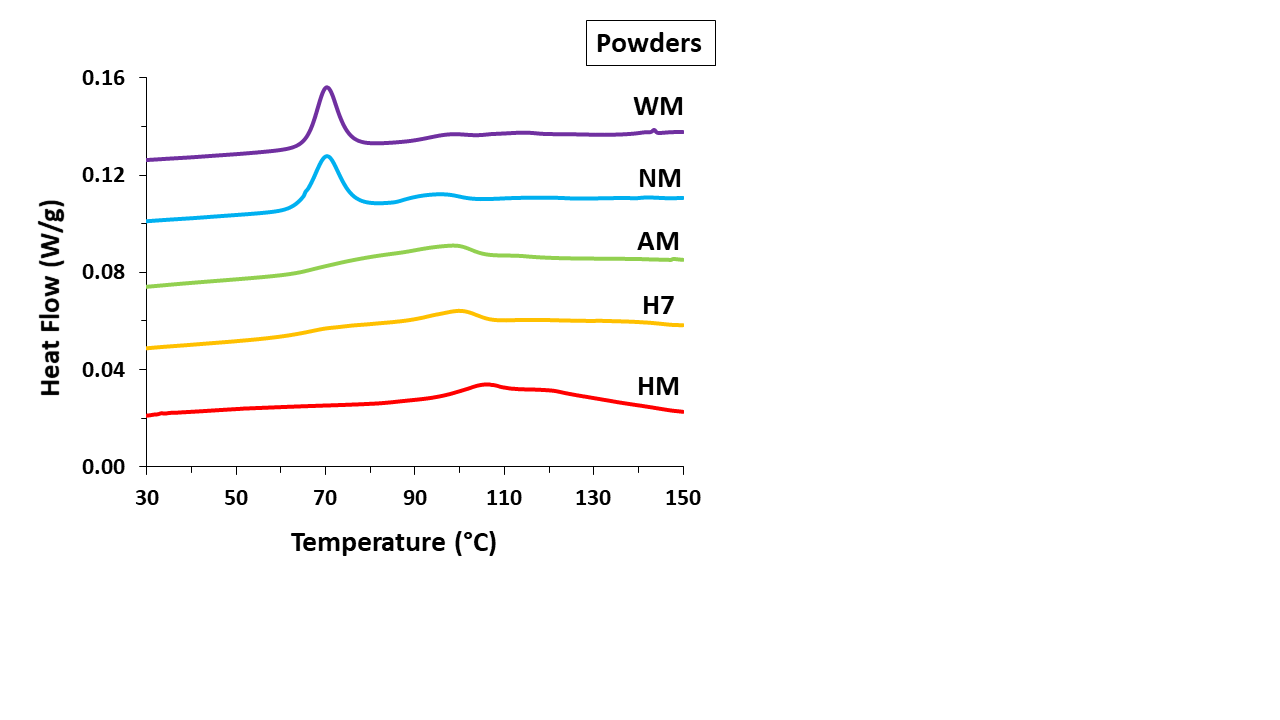 | 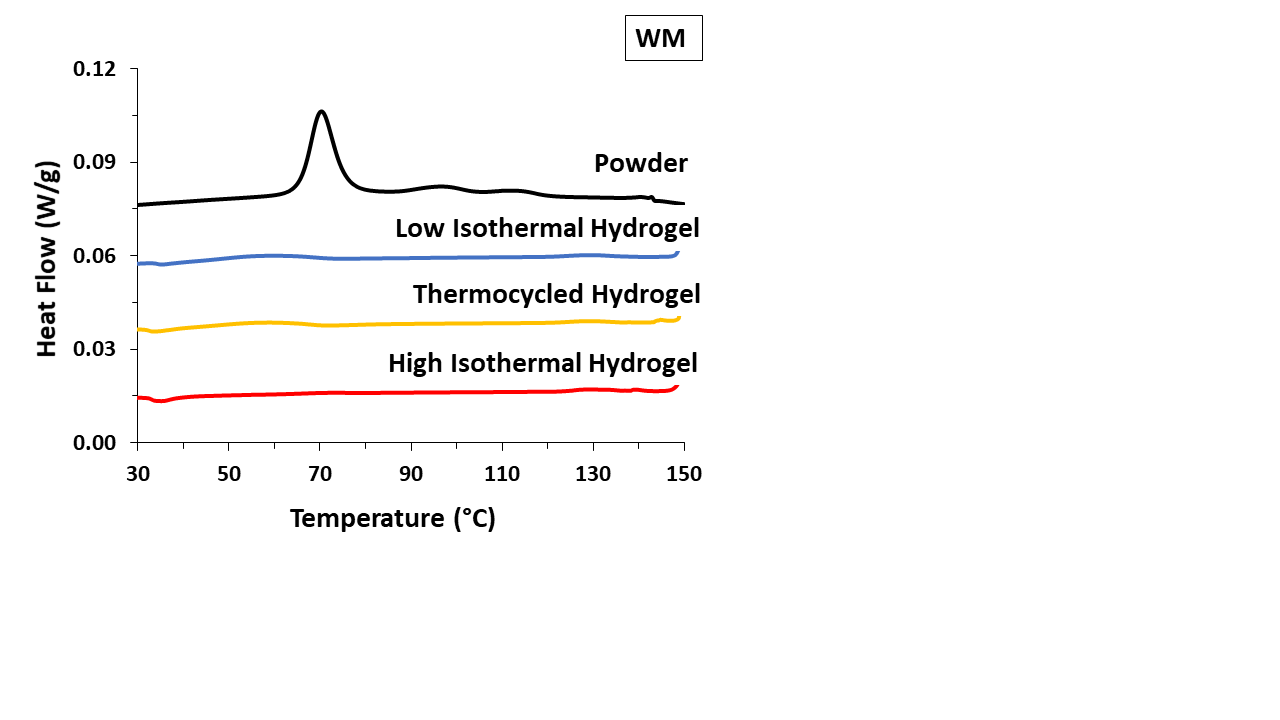 |
| --- | --- |
| 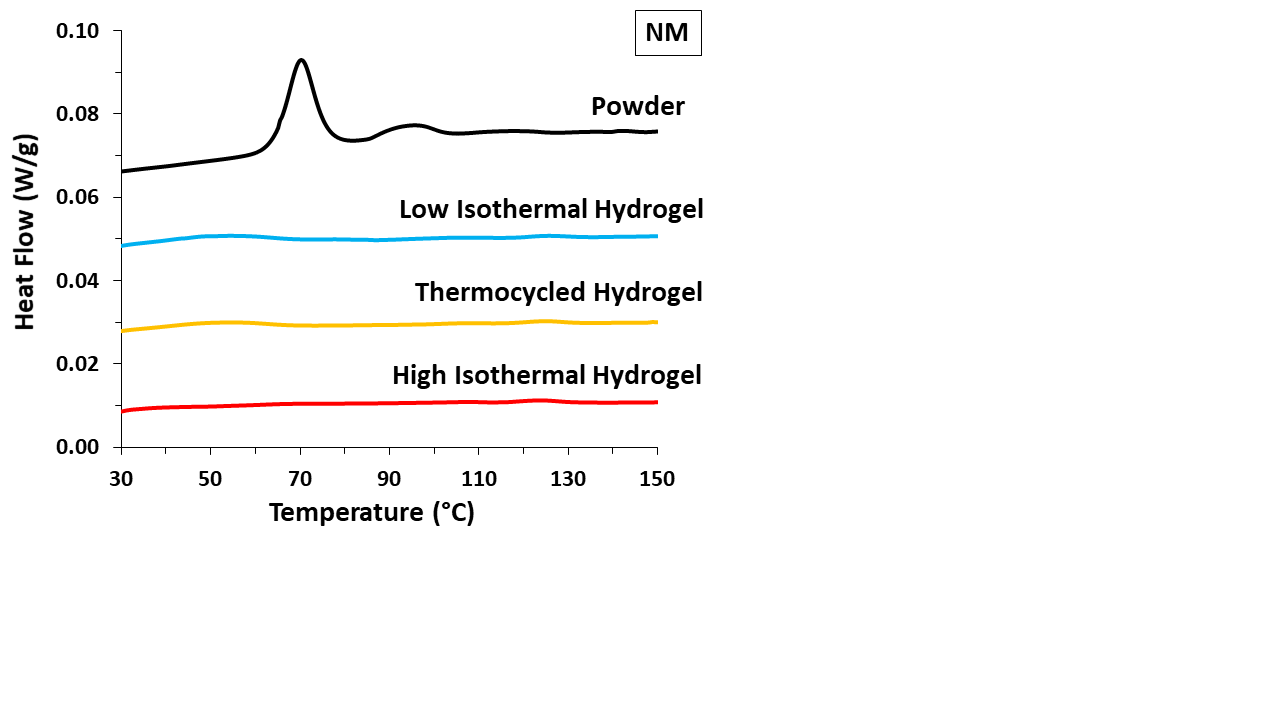 | 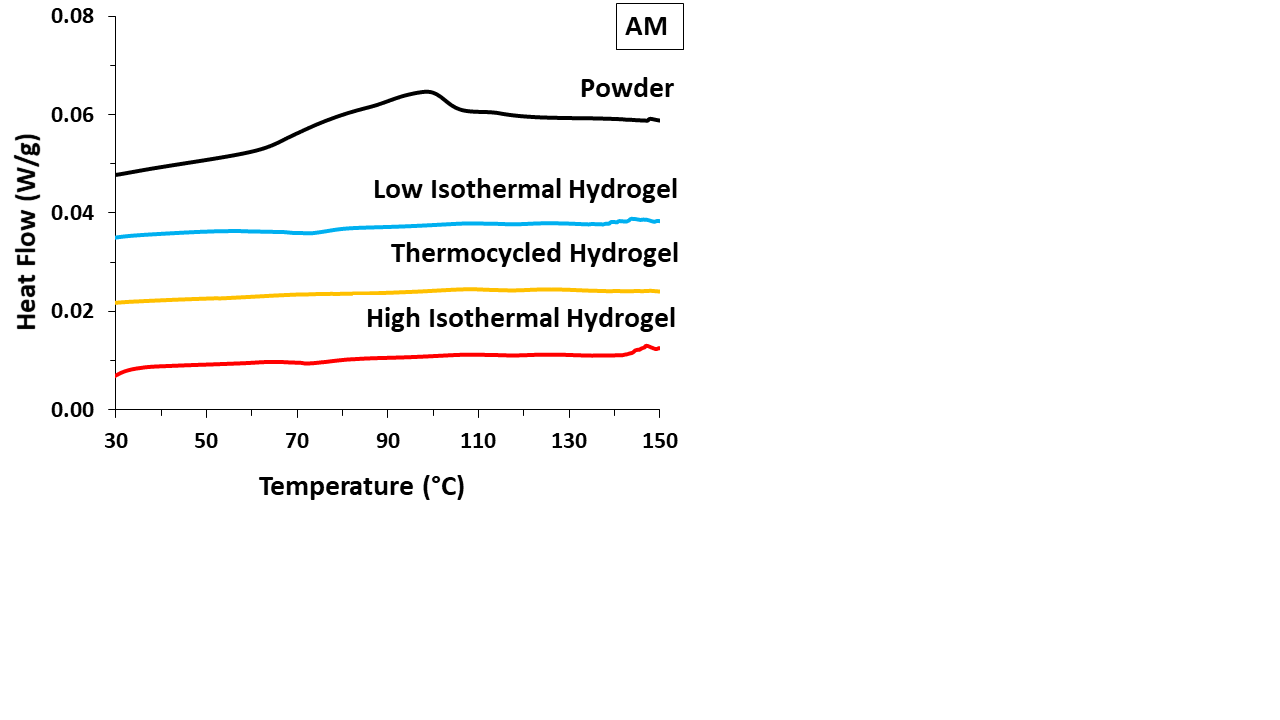 |
| 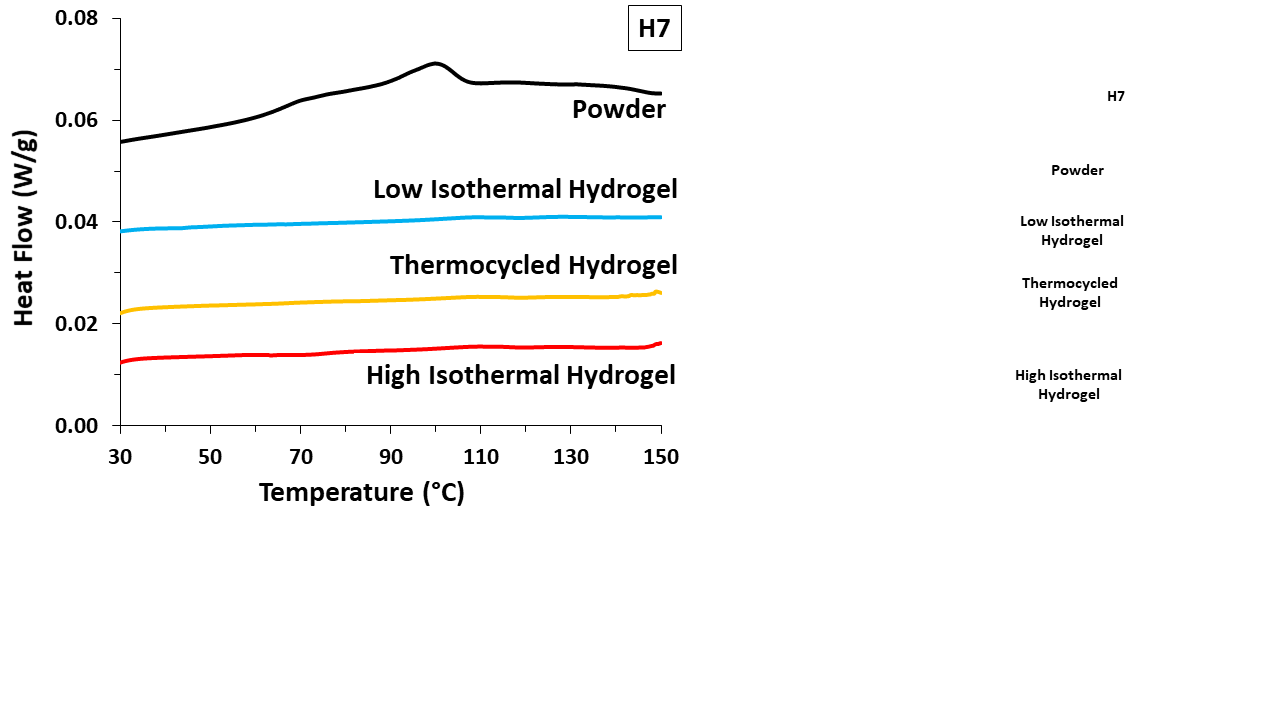 | 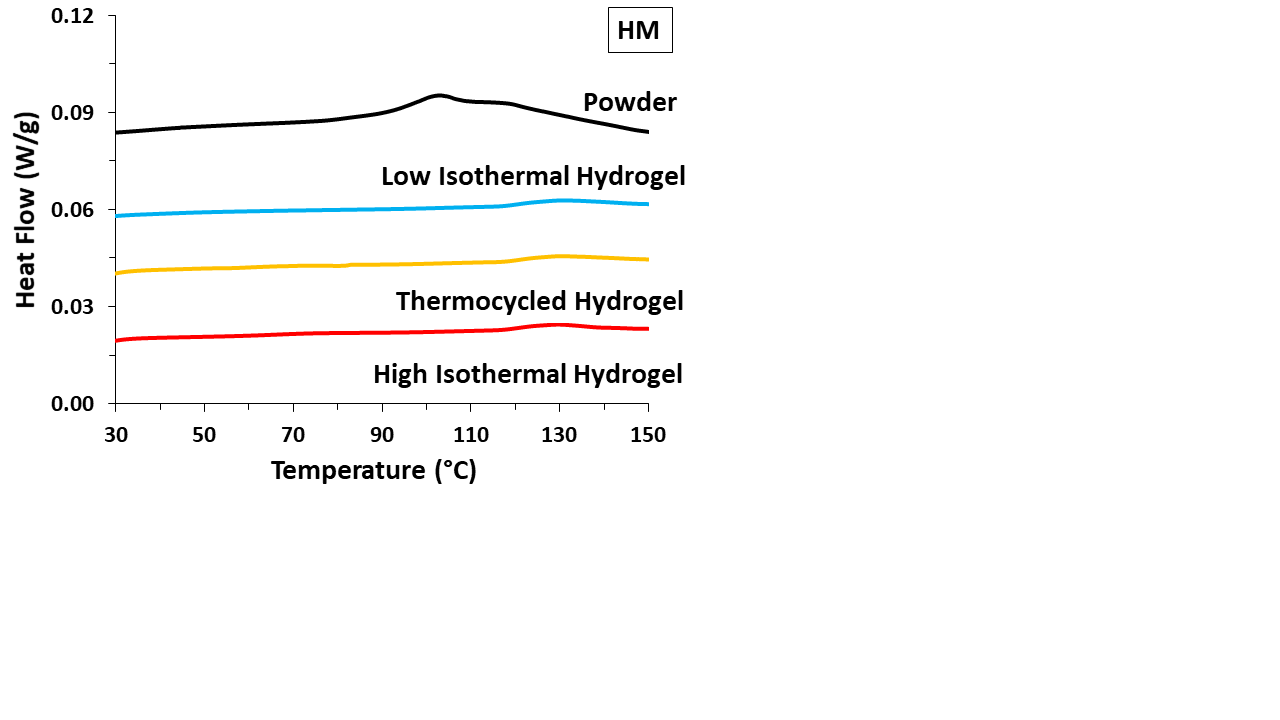 |

*Figure S5: Composite DSC thermograms of waxy maize (WM), normal maize (NM), amylomaize (AM), Hylon VII™ (H7) and Hi-Maize 260™ (HM) starch powders, and their corresponding hydrogels prepared under different storage conditions. Curves have been offset for visual clarity purposes.*

**Powders**

*Table S1: Gelatinisation parameters (onset temperature, T_O_, melting or peak temperature, T_M_, conclusion temperature, T_C_, endothermic breadth, T_C_ – T_O_ and enthalpy of transition, ΔH) of all starch powders, with units given in parentheses.*


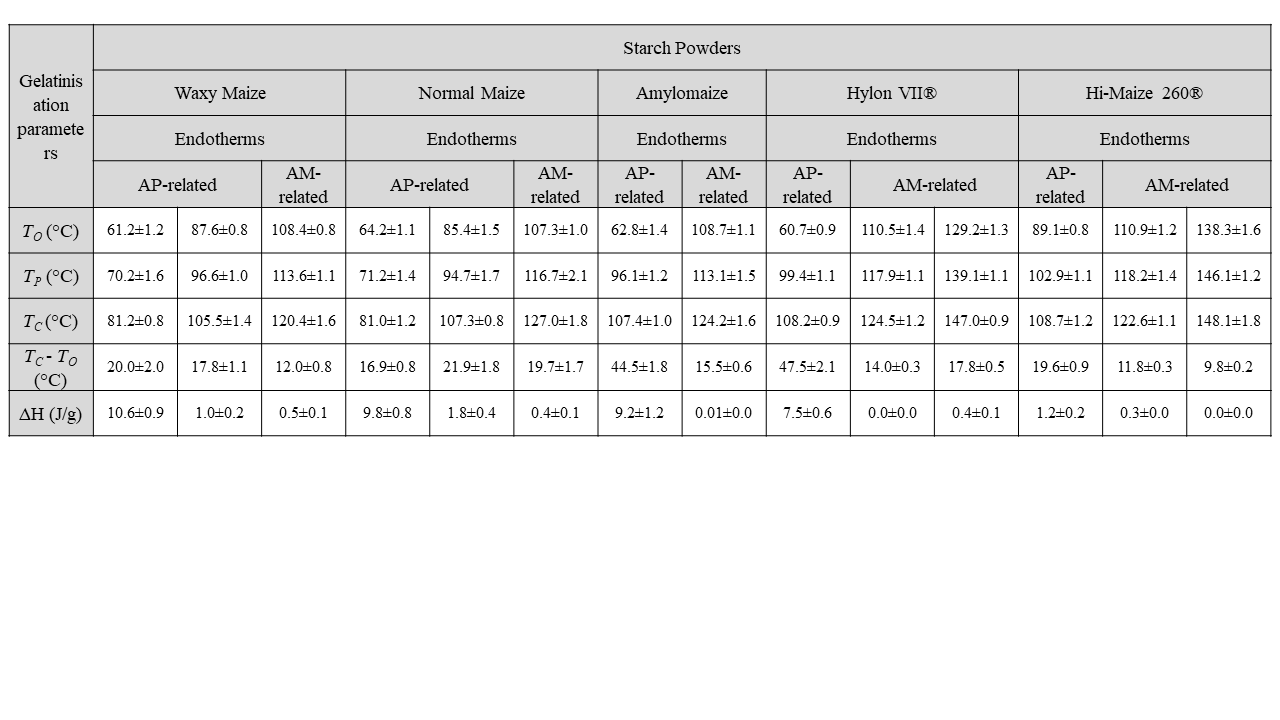


*Table S2: Gelatinisation parameters (onset temperature, T_O_, melting or peak temperature, T_M_, conclusion temperature, T_C_, endothermic breadth, T_C_ – T_O_ and enthalpy of transition, ΔH) of all low isothermal starch hydrogels, with units given in parentheses.*


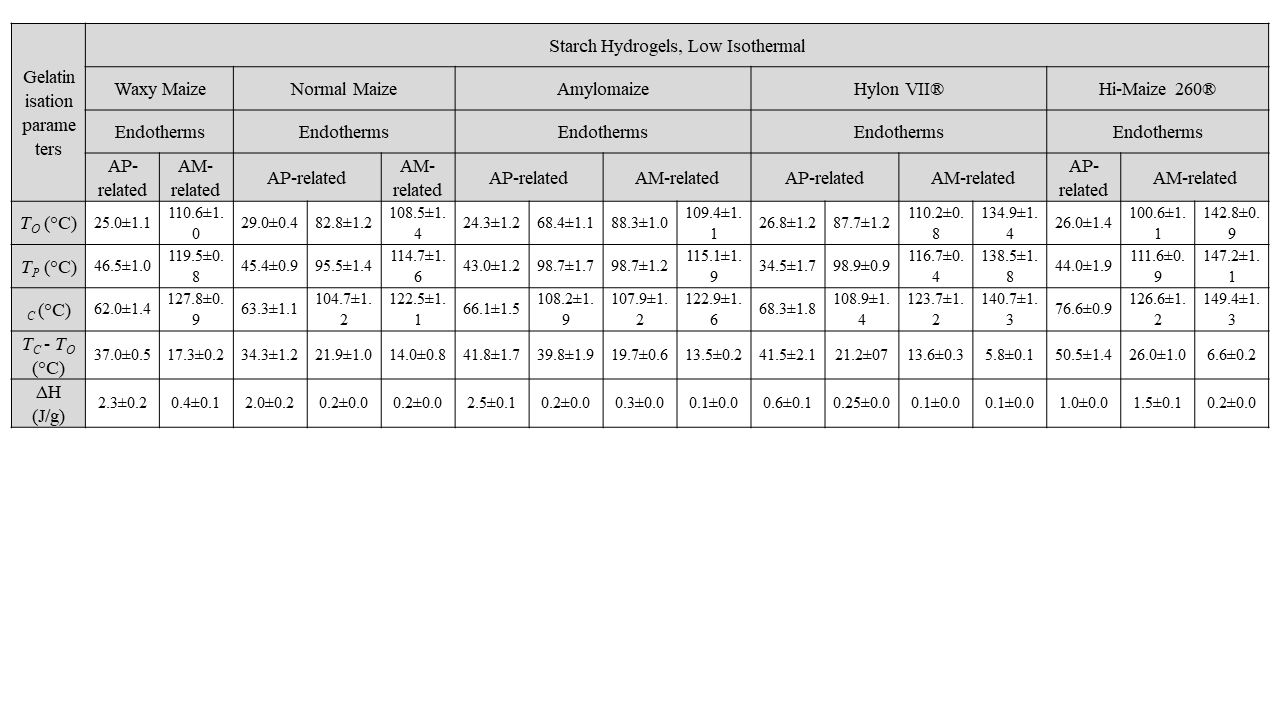


*Table S3*: *Gelatinisation parameters (onset temperature, T_O_, melting or peak temperature, T_M_, conclusion temperature, T_C_, endothermic breadth, T_C_ – T_O_ and enthalpy of transition, ΔH) of all thermocycled starch hydrogels, with units given in parentheses.*


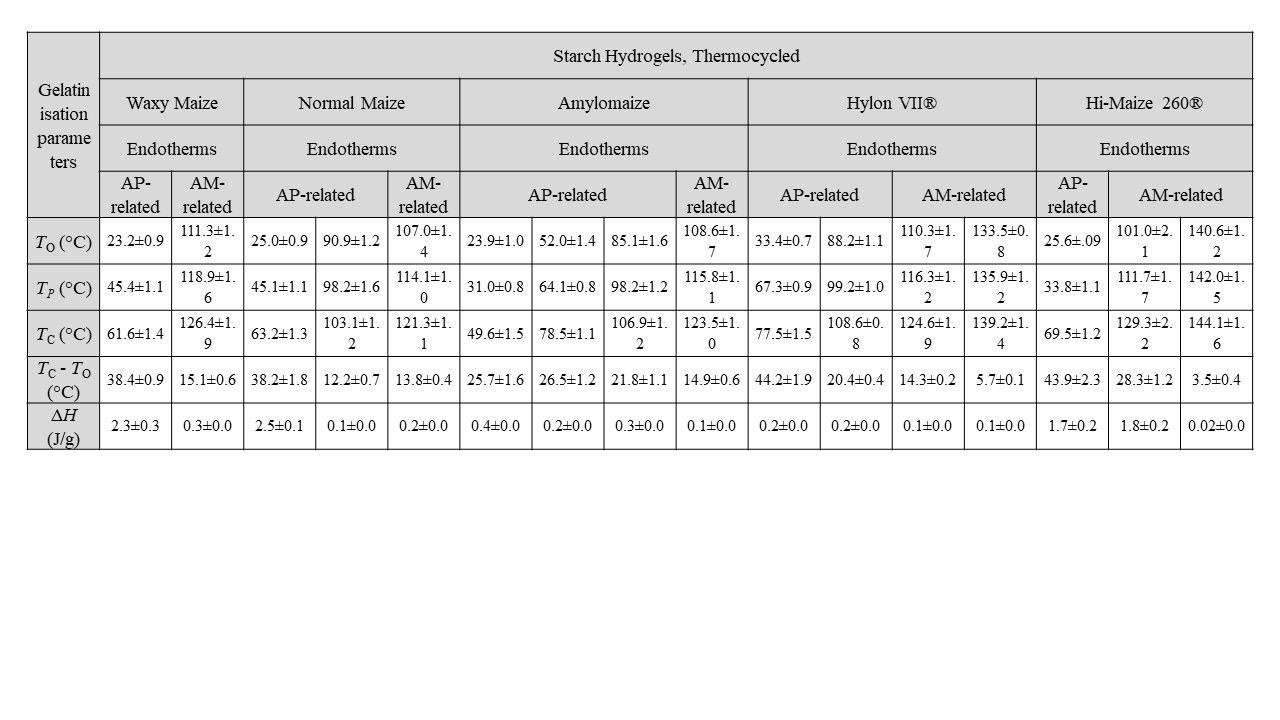


*Table S4:* *Gelatinisation parameters (onset temperature, T_O_, melting or peak temperature, T_M_, conclusion temperature, T_C_, endothermic breadth, T_C_ – T_O_ and enthalpy of transition, ΔH) of all high isothermal starch hydrogels, with units given in parentheses.*


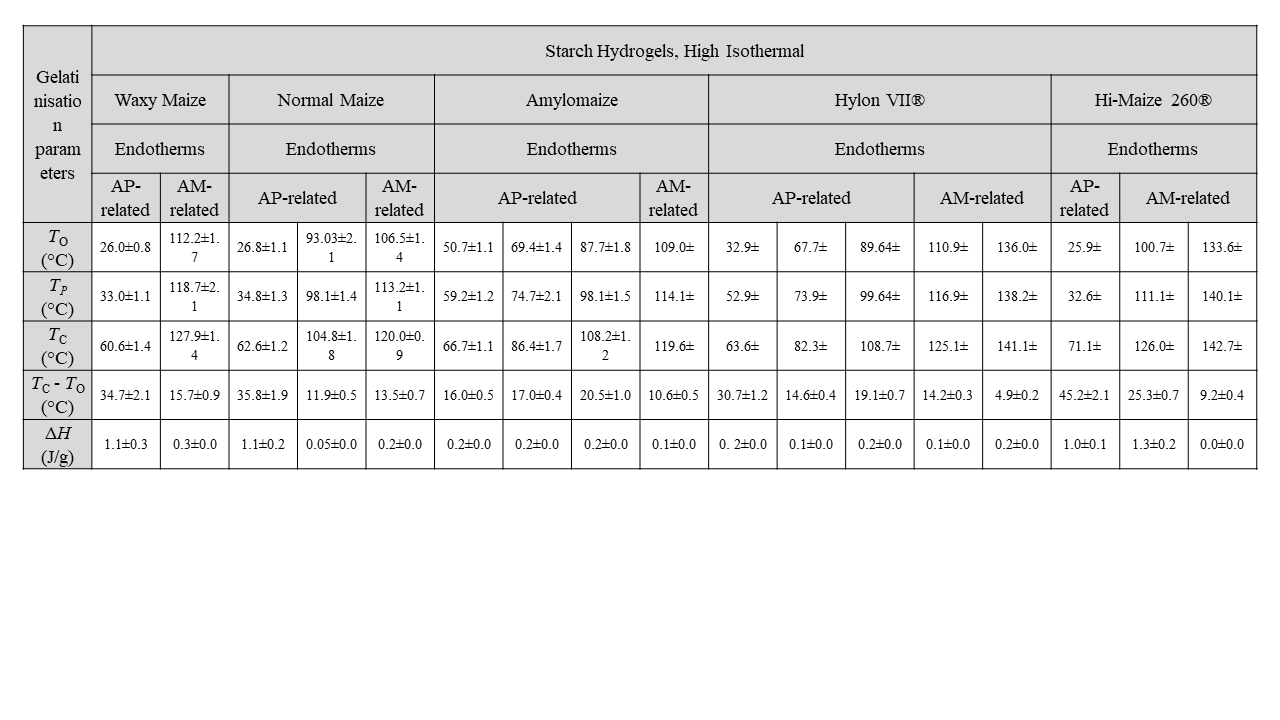


*Table S6:* *Sub-ambient exothermic transition parameters (onset temperature, T_O_, concluding temperature, T_C_ and enthalpy of transition, ΔH) of all low temperature isothermally stored starch hydrogels, with units given in parentheses.*

| Starch Type | Cooling Cycle, 5 °C/min | | |
| --- | --- | --- | --- |
|  | Onset Point (°C) | Concluding Point (°C) | ΔH (J/g) |
| Waxy Maize | -15.66 | -25.31 | 189.63 |
| Normal Maize | -13.73 | -23.95 | 212.86 |
| Amylomaize | -17.98 | -25.63 | 182.35 |
| Hylon VII™ | -15.58 | -24.73 | 195.94 |
| Hi-Maize 260 | -15.77 | -22.80 | 137.82 |

1. **Rheology**


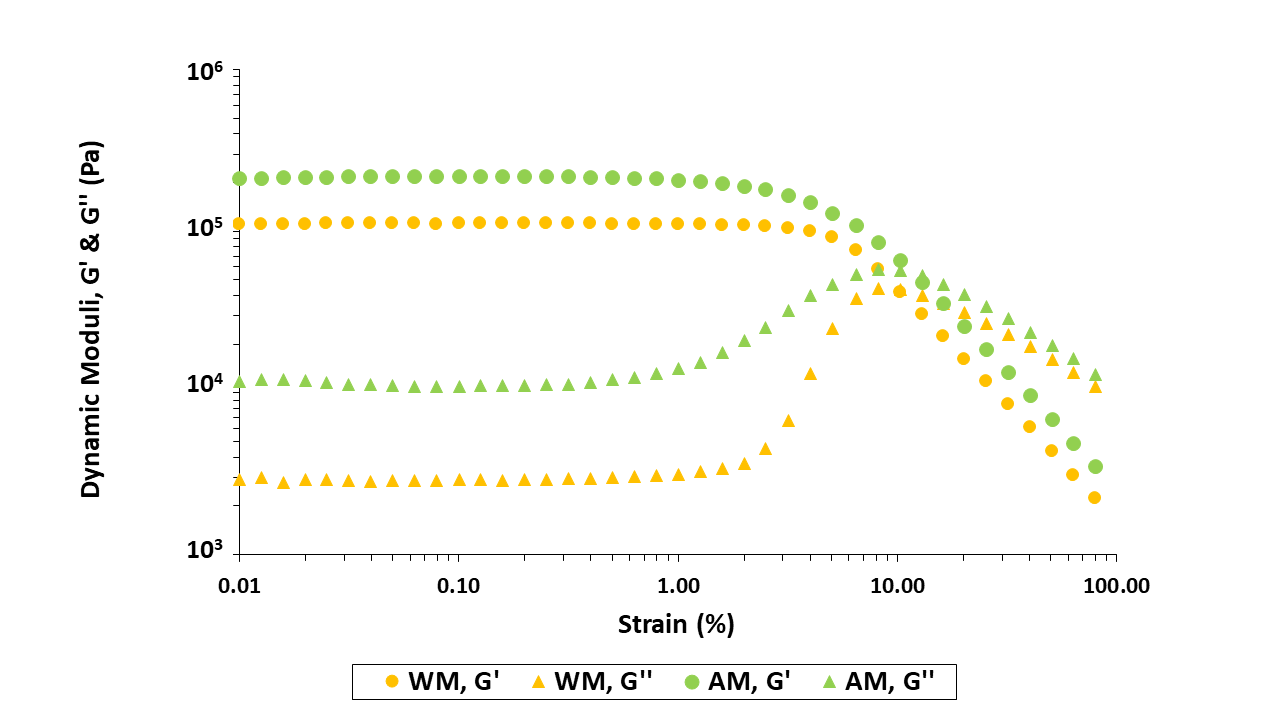


*Figure S6: Storage and loss moduli (G’ and G’’, respectively) of waxy and amylomaize starch hydrogels stored at low temperature isothermal conditions, as a function of applied strain (%). All results are expressed in Pa and are the average of a minimum of three repeats.*


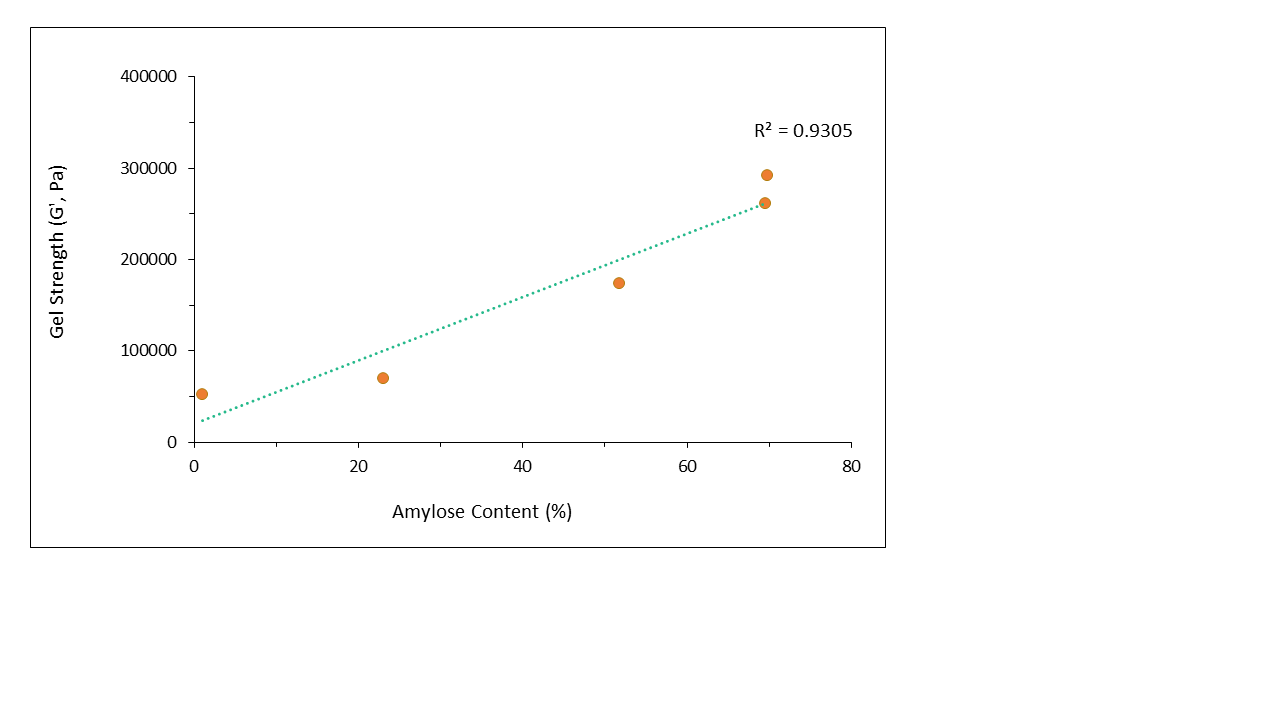


*Figure S7: Linear correlation plot between maize starch hydrogels’ strength (G’, Pa) and respective amylose content (%), with displayed trendline and R^2^ value.*

1. **Powder X-ray Diffraction (PXRD)**


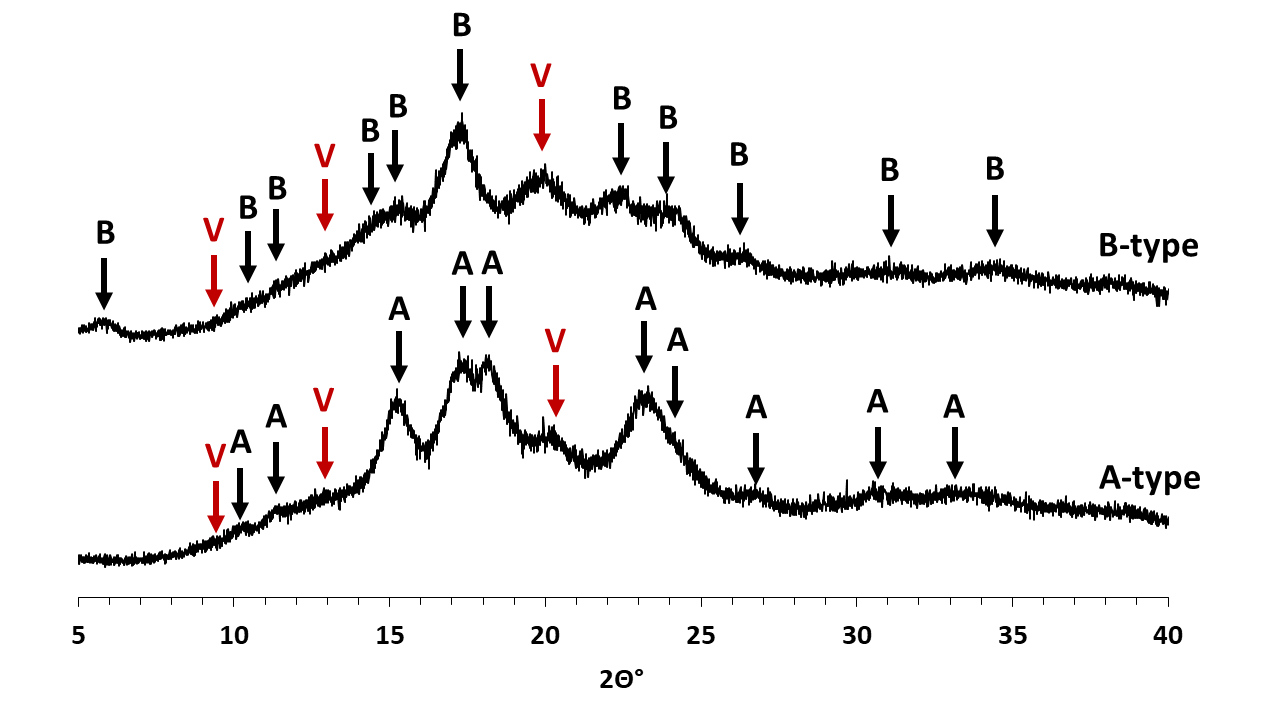


*Figure S3: Powder X-ray Diffraction pattern of A- and B-type powder starches (normal and Hylon VII™ maize powders shown here, respectively), with black arrows indicating the peaks (10 for A-type and 11 for B-type) associated with A- and B-type type crystallinity chosen for peak fitting, and red arrows indicating the peaks associated with V-type amylose arrangements.*


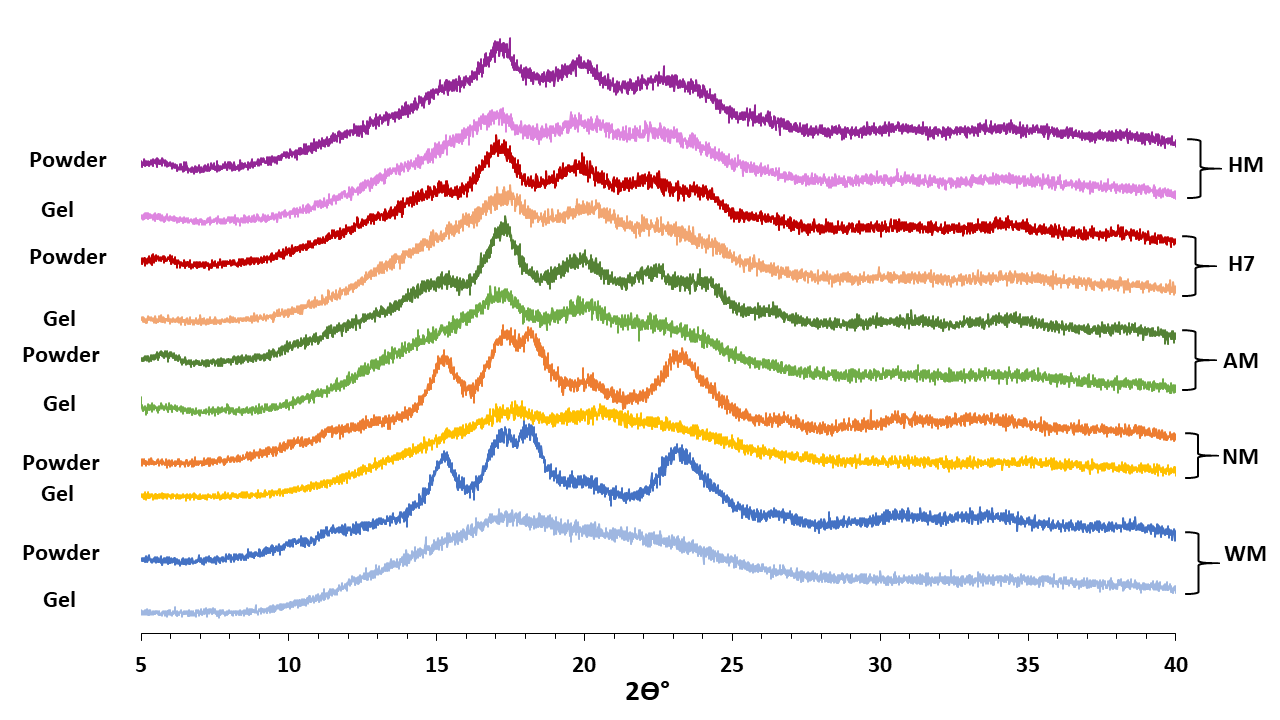


*Figure S8: Powder X-ray Diffraction patterns of waxy maize (WM), normal maize (NM), amylomaize (AM), Hylon VII™ (H7) and Hi-Maize 260™ (HM) powder samples and their respective hydrogels prepared at low temperature isothermal conditions. Diffractograms have been displaced for visual clarity purposes.*


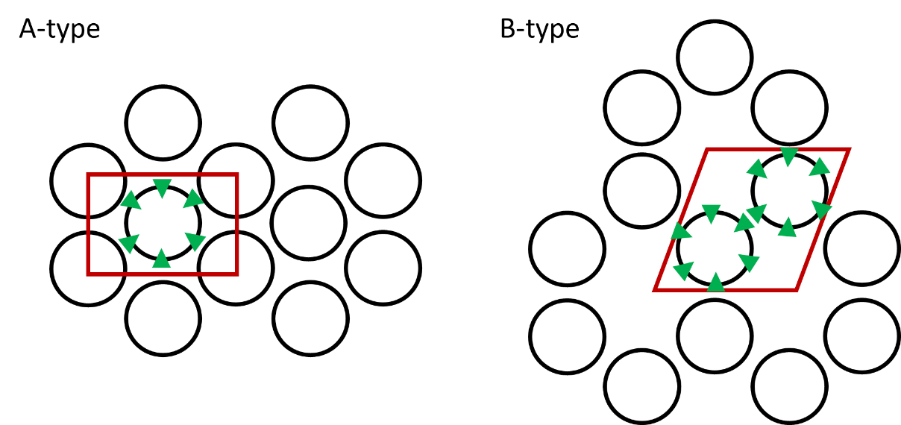


*Figure S9: Diagram of helical packing organisation and unit cells (red) in A- and B-type starches, with each circle representing a through-centre view of double helices. Possible positions of C-1 nuclei within a unit cell are given in green (adapted from the works of Gidley & Bociek* (Gidley, 1985) *).*

1. **Nuclear Magnetic Resonance (NMR) Spectroscopy**


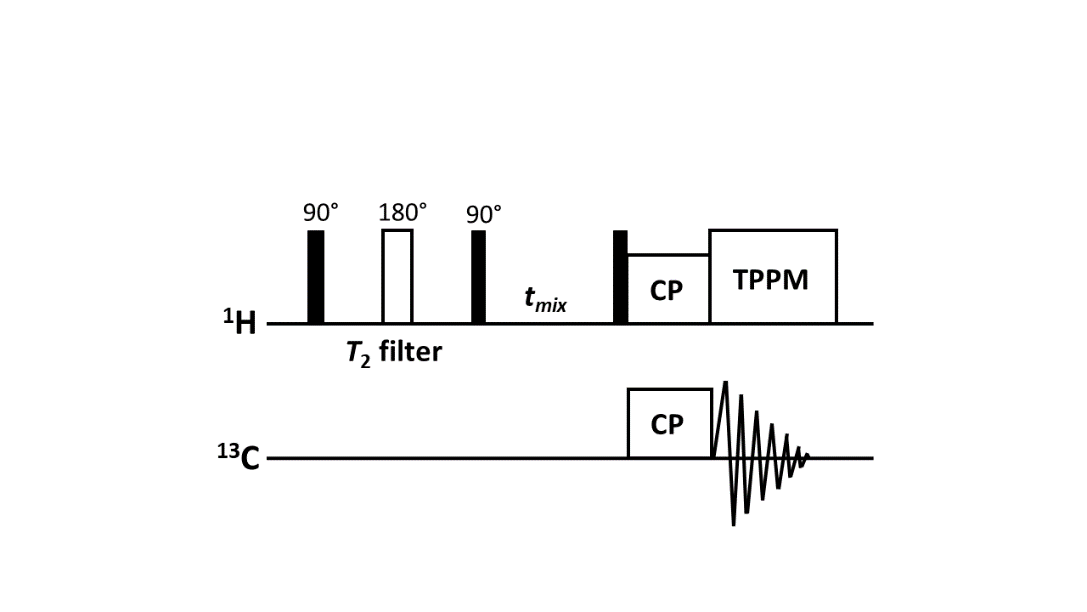


*Figure S4: Water Polarisation Transfer-Cross Polarisation (WPT-CP) pulse sequence.*

*Table S5: Assignment of carbon site resonances in all maize starch solid powders analysed, in accordance with published literature available.*


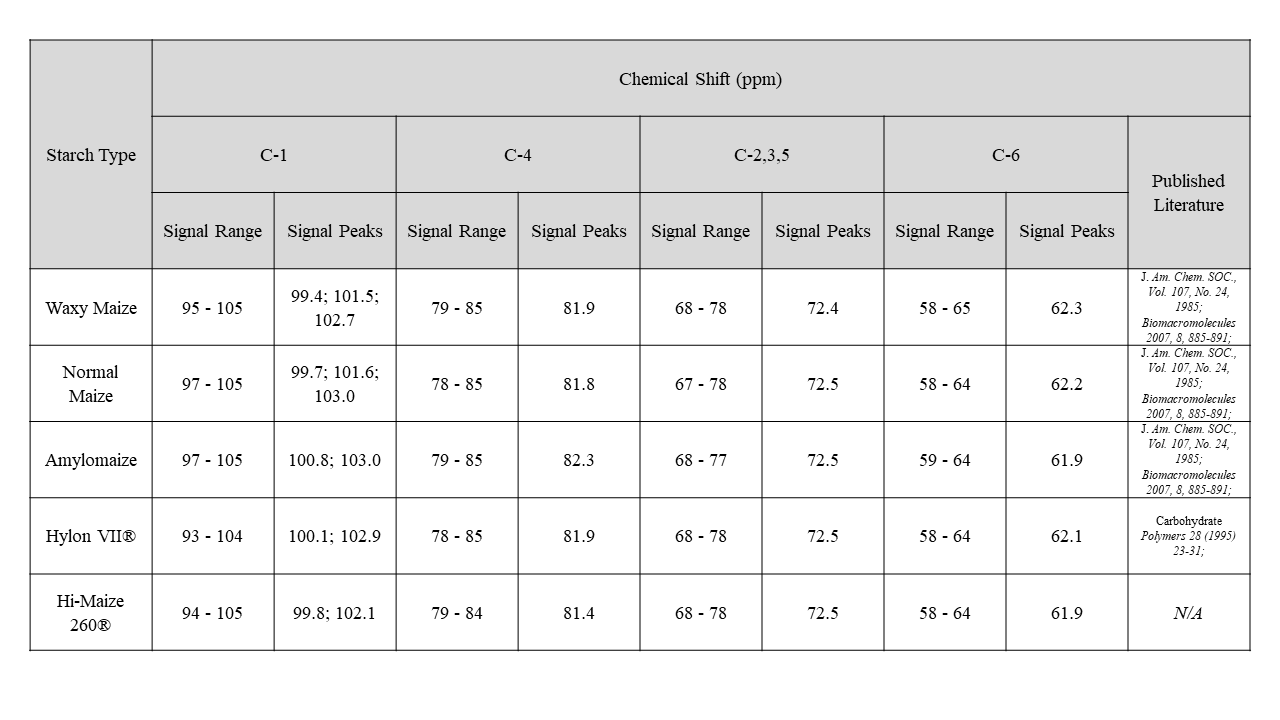


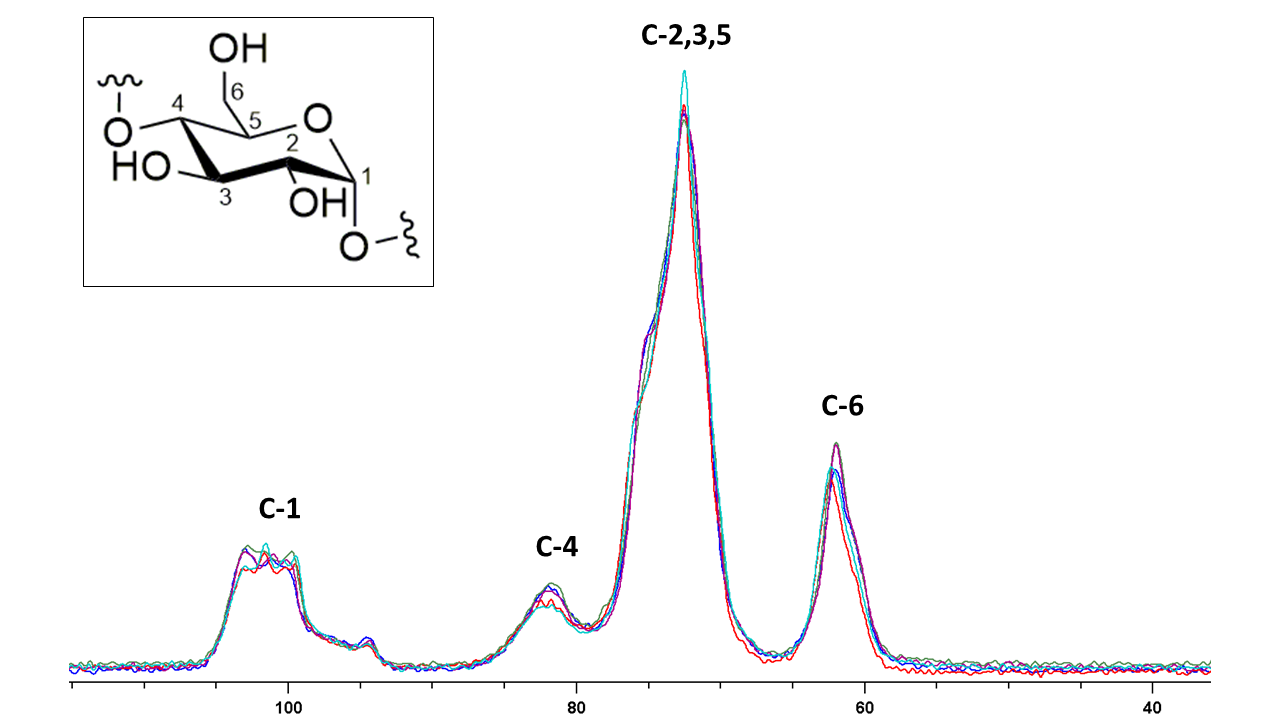


*Figure S10: ^13^C CP/MAS NMR spectra of waxy maize (teal), normal maize (red), amylomaize (magenta), Hylon VII™ (blue) and Hi-Maize 260™ (green) powder samples, with inlay showing the glucose monomer with ^13^C atomic numbering*.


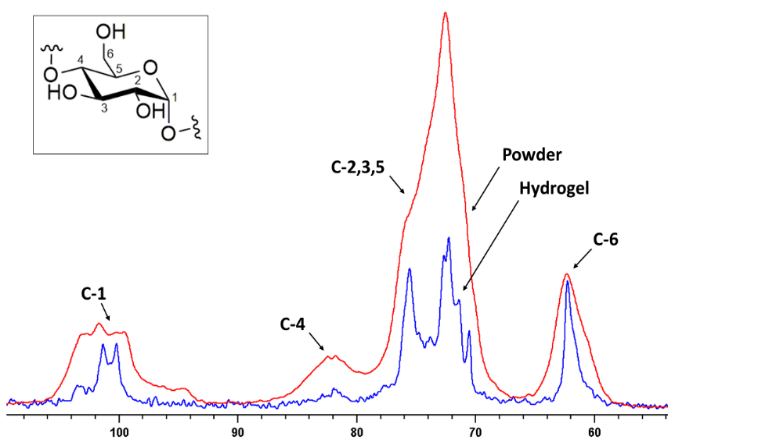


*Figure S11: ^1^H-^13^C CP/MAS NMR spectra of normal maize powder (red) and low temperature isothermally stored hydrogel (blue) with inlay showing the glucose monomer with ^13^C atomic numbering, and arrows pointing towards each of the 4 peak regions attributed to ^13^C nuclei in the starch monomer.*


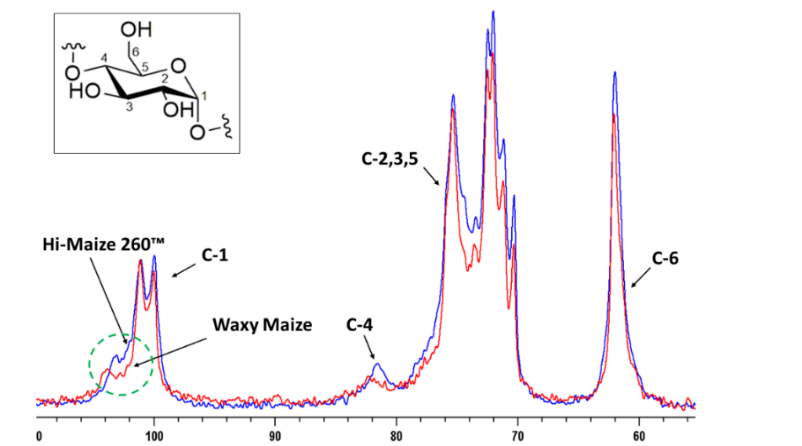


*Figure S12: ^1^H-^13^C CP/MAS NMR spectra of waxy maize (red) and Hi-Maize 260™ (blue) low temperature isothermally stored hydrogels, where area in green shows the 100‑104 ppm regions ascribed to V‑type amylose and amorphous starch content. Inlay showing the glucose monomer with ^13^C atomic numbering, and arrows pointing towards each of the 4 peak regions attributed to ^13^C nuclei in the starch monomer.*

| 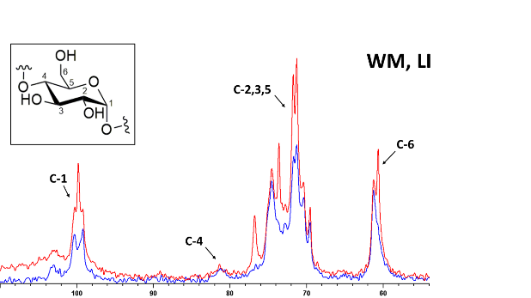 | 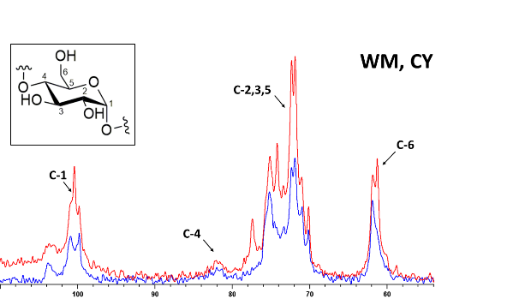 | 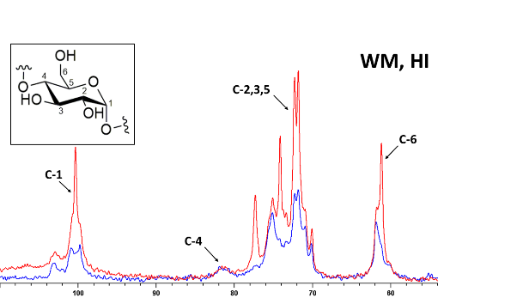 |
| --- | --- | --- |
| 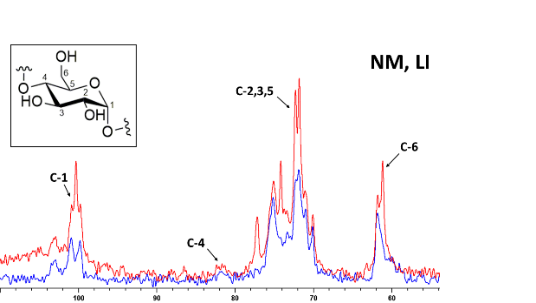 | 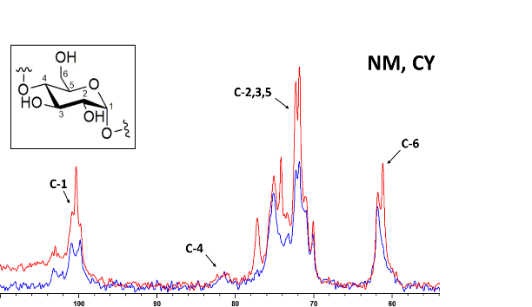 | 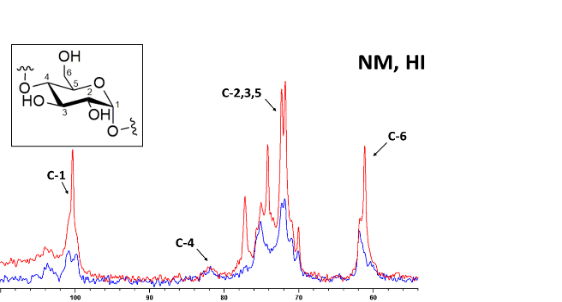 |
| 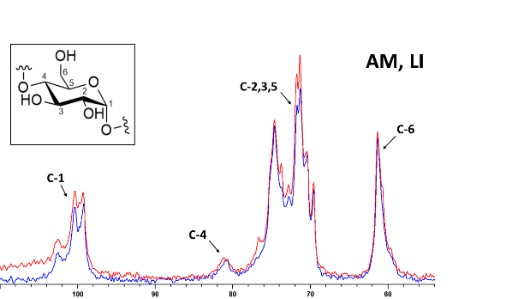 | 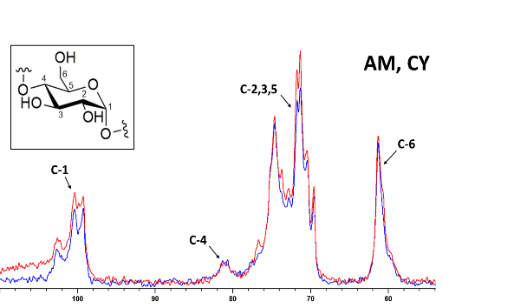 | 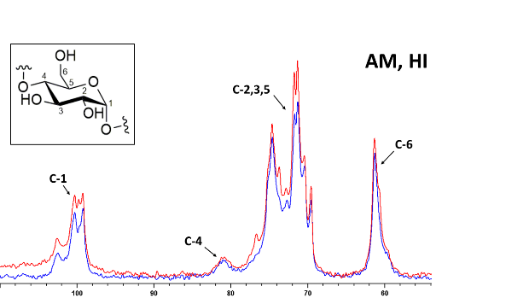 |
| 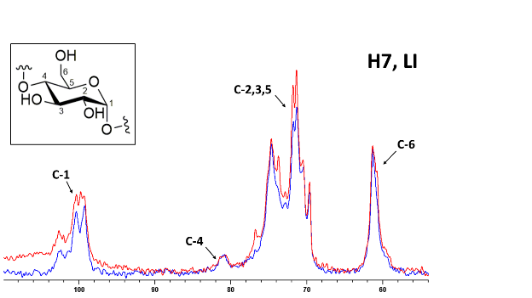 | 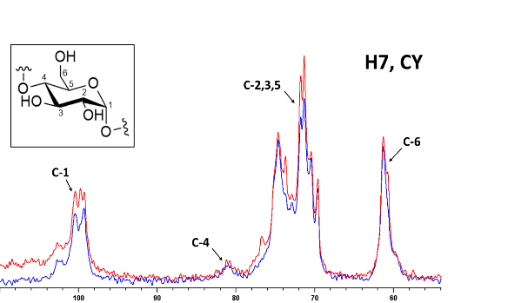 | 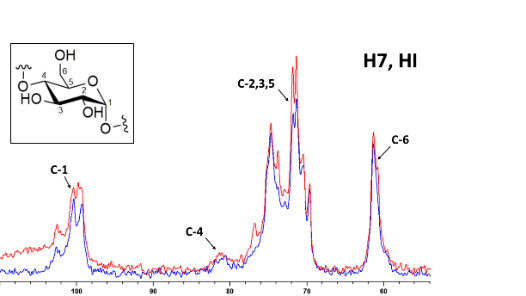 |
| 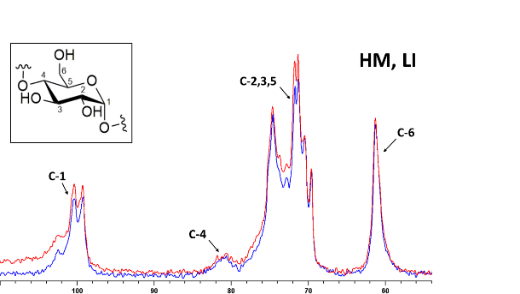 | 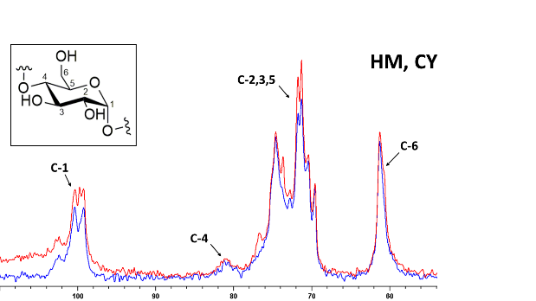 | 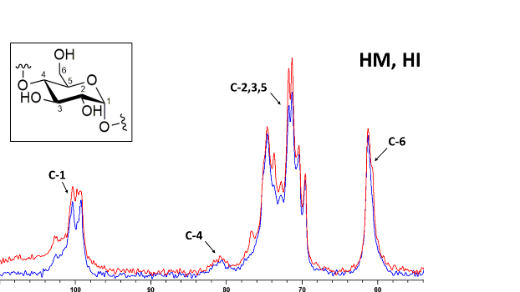 |

*Figure S13: ^13^C CP and CPSP/MAS NMR spectra (blue and red, respectively) of waxy maize (WM), normal maize (NM), amylomaize (AM), Hylon VII™ (H7) and Hi-Maize 260™ (HM) hydrogels, prepared in three different conditions – low isothermal (LI), thermocycled (CY) and high isothermal (HI) storage. Inlays showing the glucose monomer with ^13^C atomic numbering.*

| 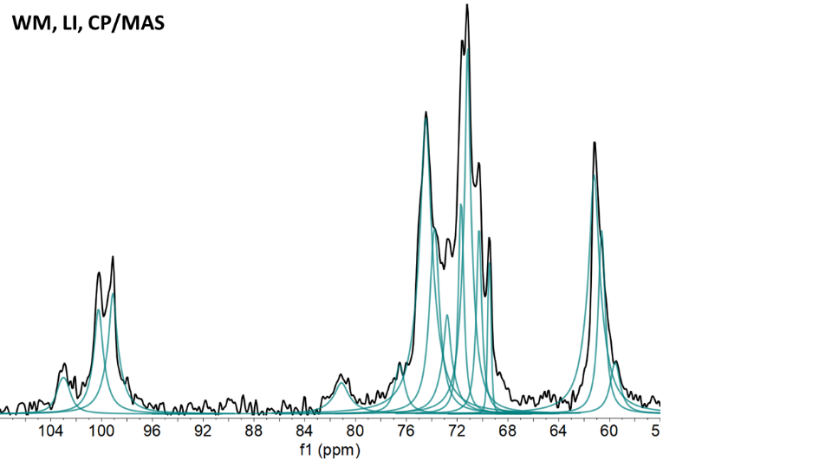 | 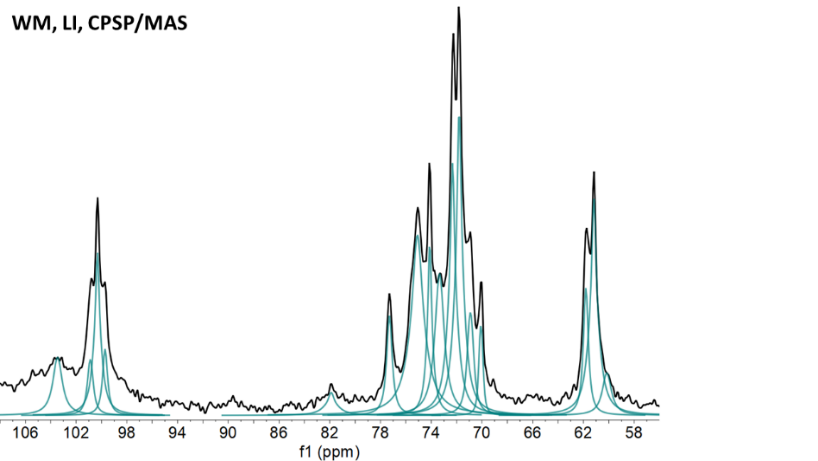 |
| --- | --- |
| 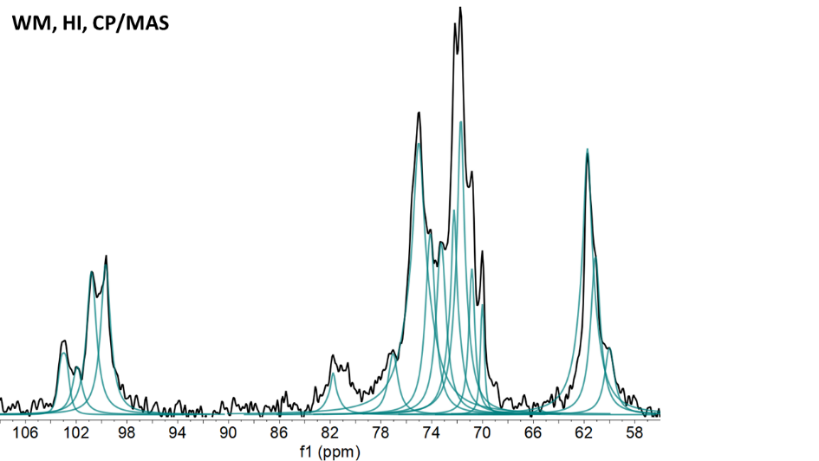 | 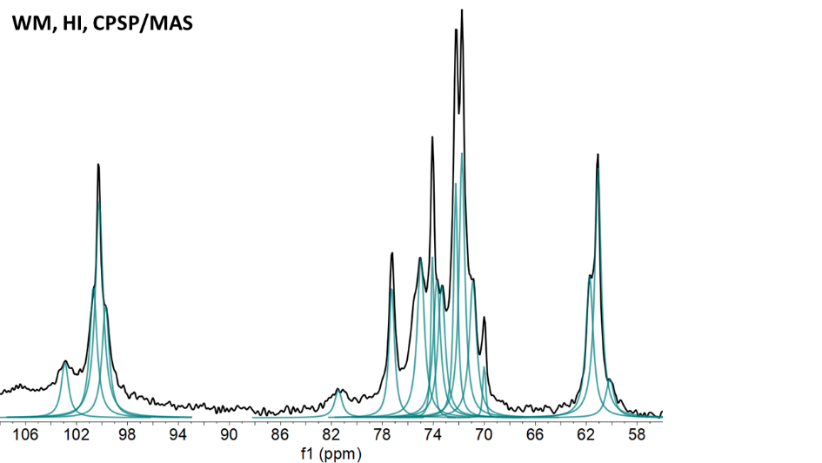 |
| 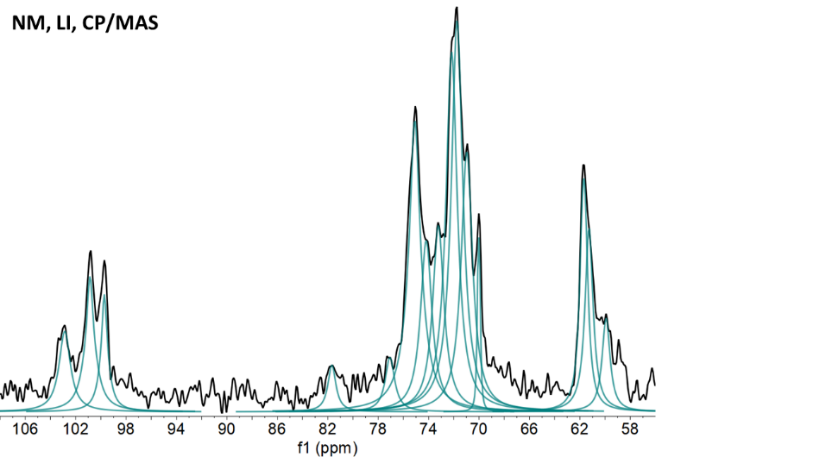 | 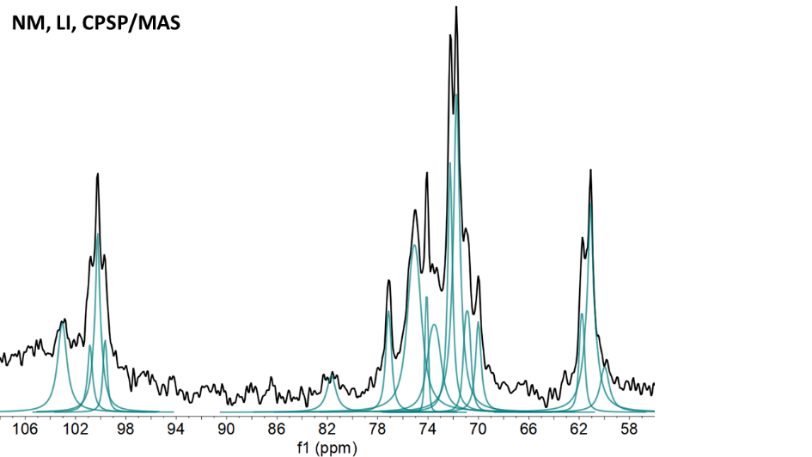 |
| 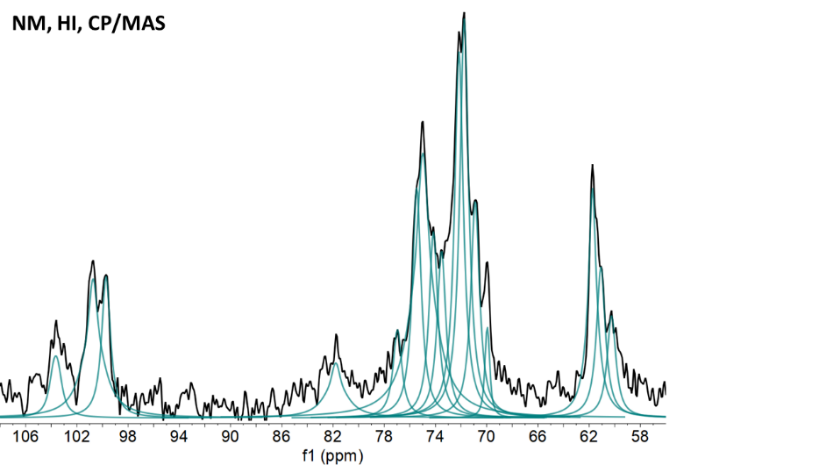 | 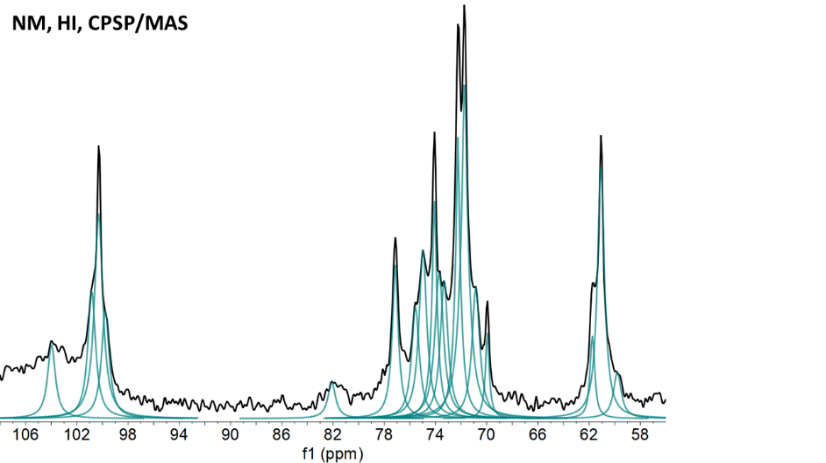 |
| 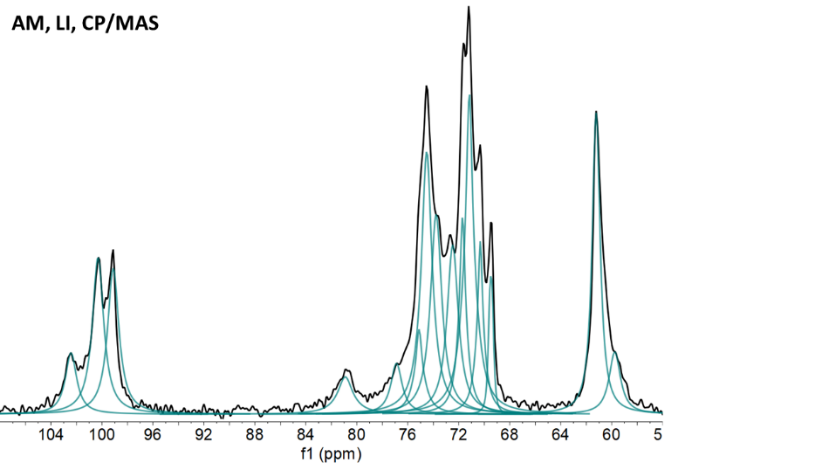 | 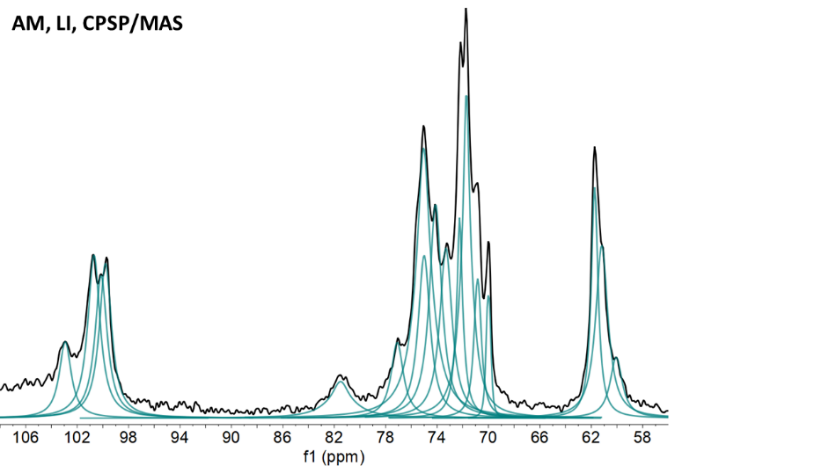 |
| 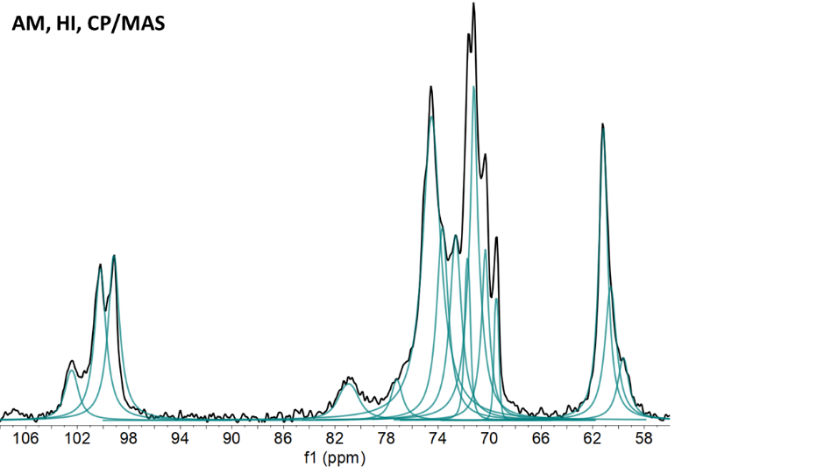 | 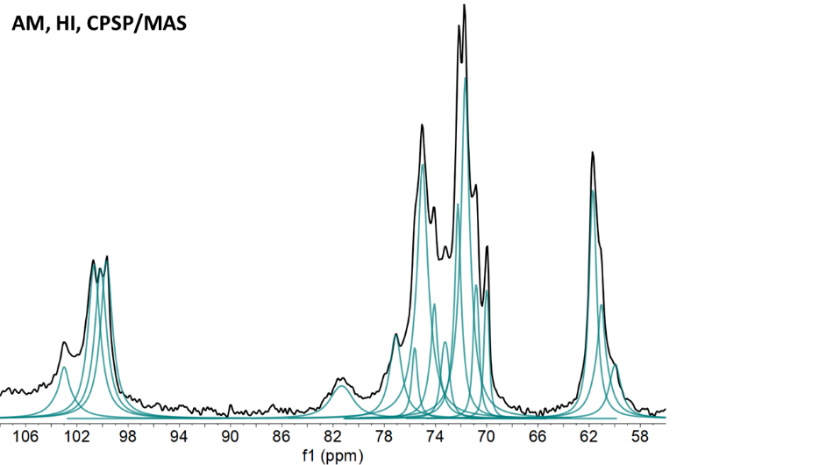 |
| 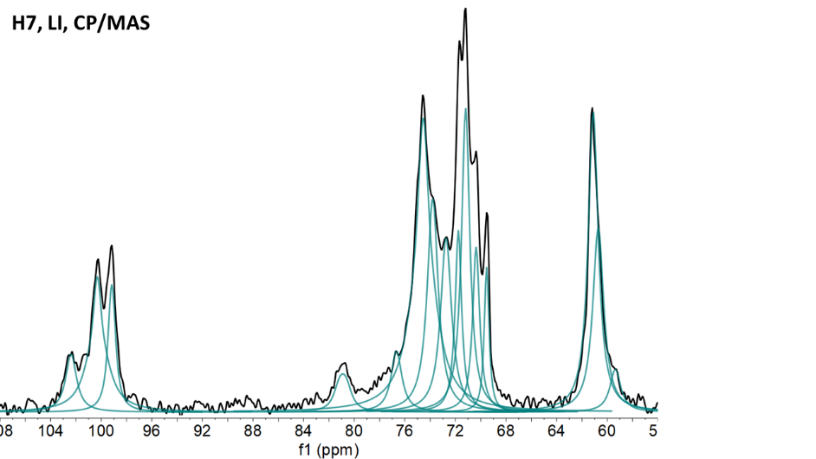 | 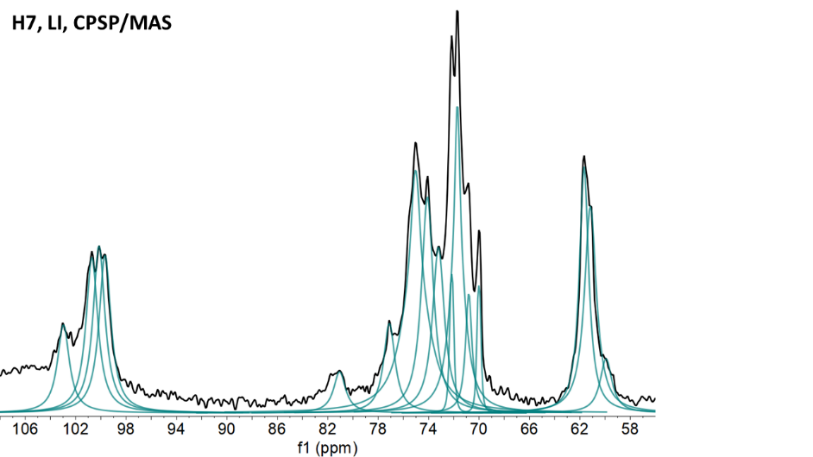 |
| 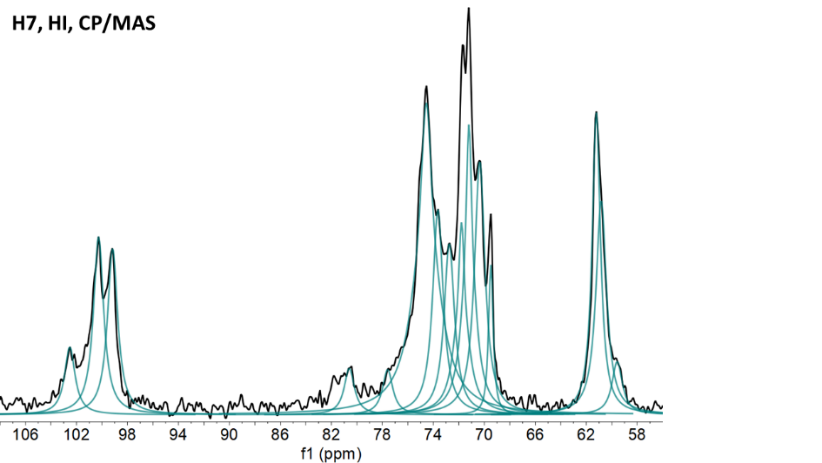 | 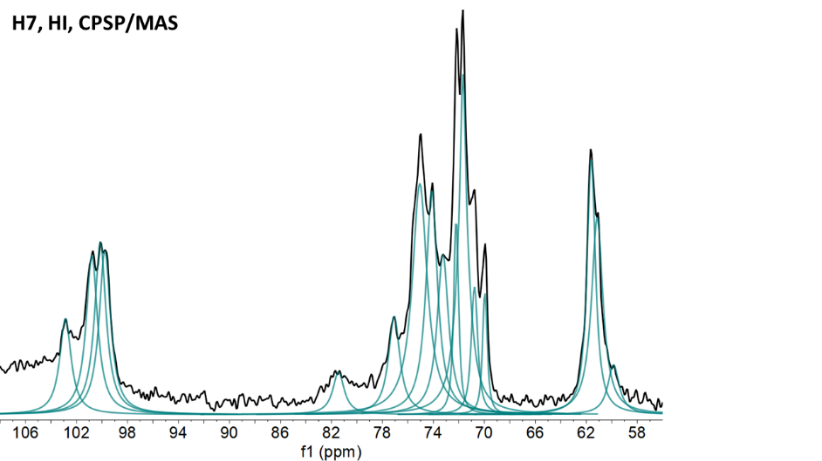 |
| 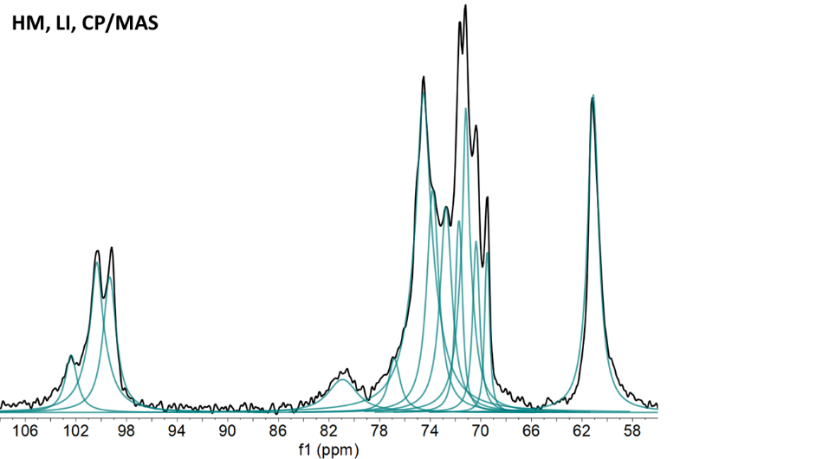 | 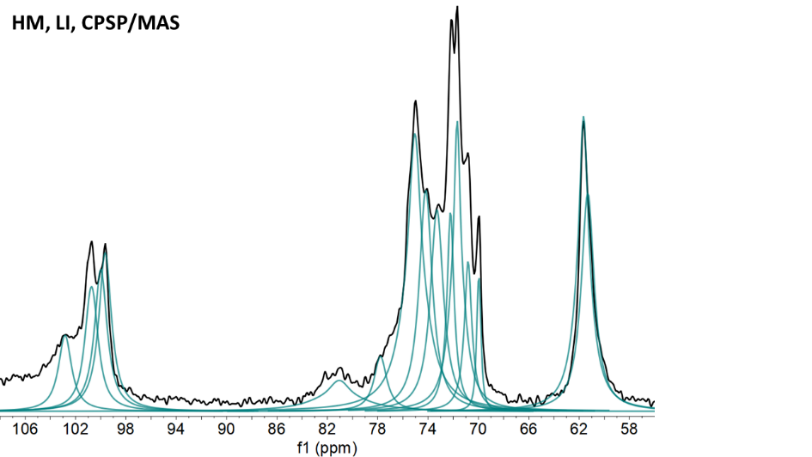 |
| 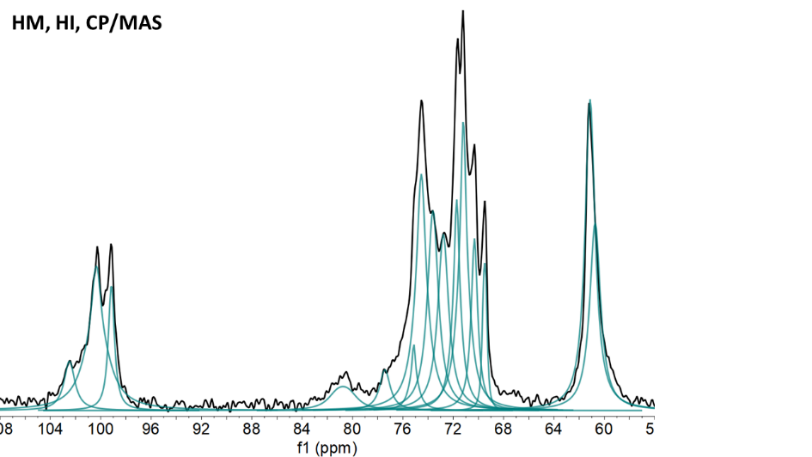 | 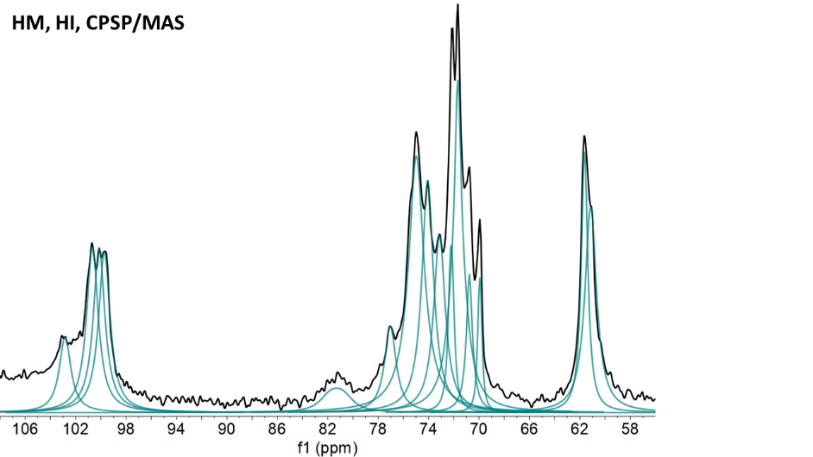 |

*Figure S14: Deconvoluted CP and CPSP/MAS (left and right, respectively) spectra of all five maize starch hydrogels, prepared under low isothermal (LI) and high isothermal (HI) conditions.*

*
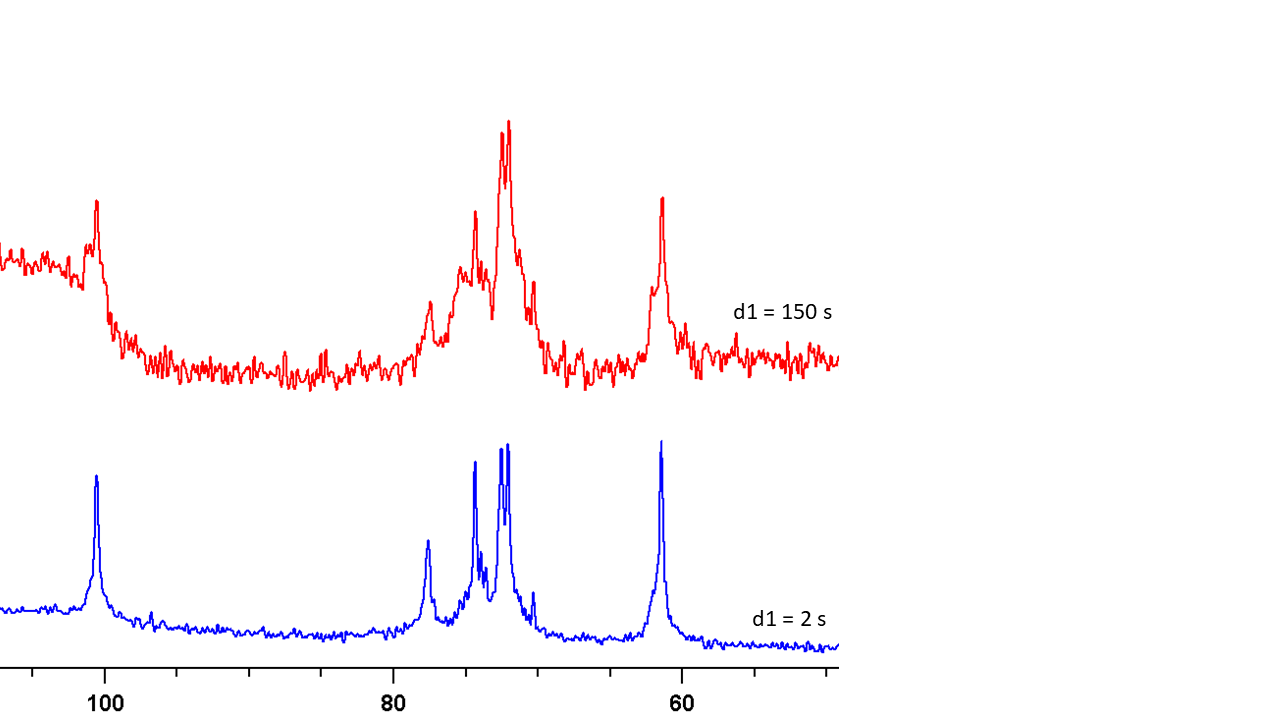
*

*Figure S15: ^13^C Direct polarisation with high power ^1^H decoupling (HPDEC) NMR spectra of low temperature isothermally stored normal maize starch hydrogels with short (blue, recycle delay of 2 s) and long (red, recycle delay of 150 s) recycle delays.*

| 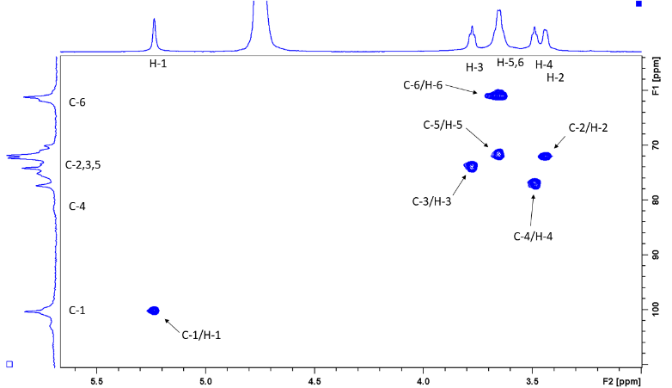 | 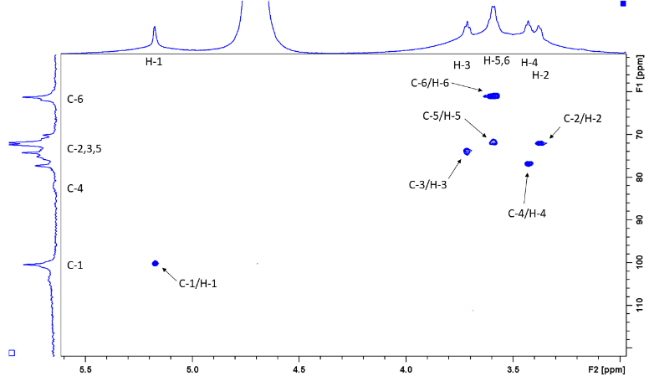 |
| --- | --- |
| 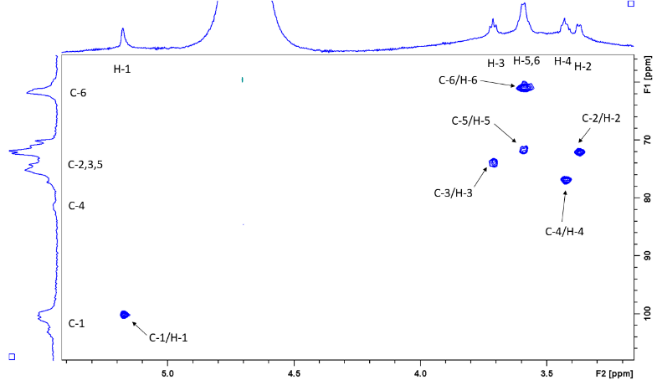 | 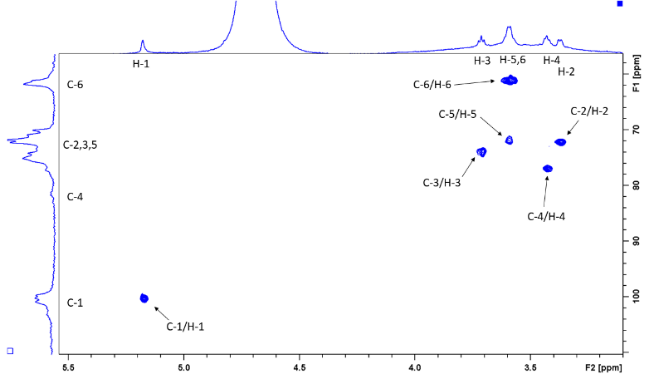 |
| *Figure S16: 1H-13C HSQC spectra of low isothermally stored starch hydrogels: waxy maize (top, left), normal maize (top, right), Hylon VII™ (bottom, left) and Hi-Maize 260™ (bottom, right), featuring peak and cross-peak assignment.* | |


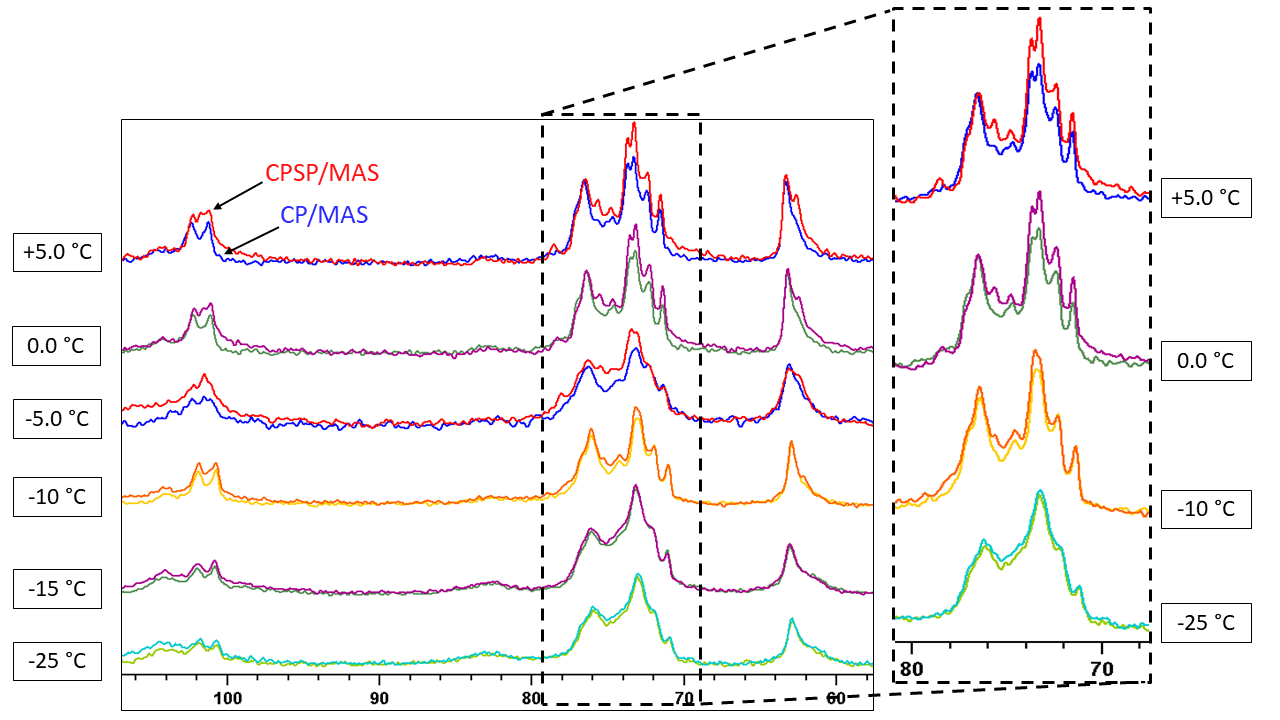


*Figure S17: Overlay ^13^C CP and CPSP/MAS NMR spectra of low isothermally stored normal maize starch hydrogels, obtained at variable temperatures, with inlay showing a zoomed in version of the C‑2,3,5 spectral region. All experiments were acquired with a minimum of 2000 scans.*


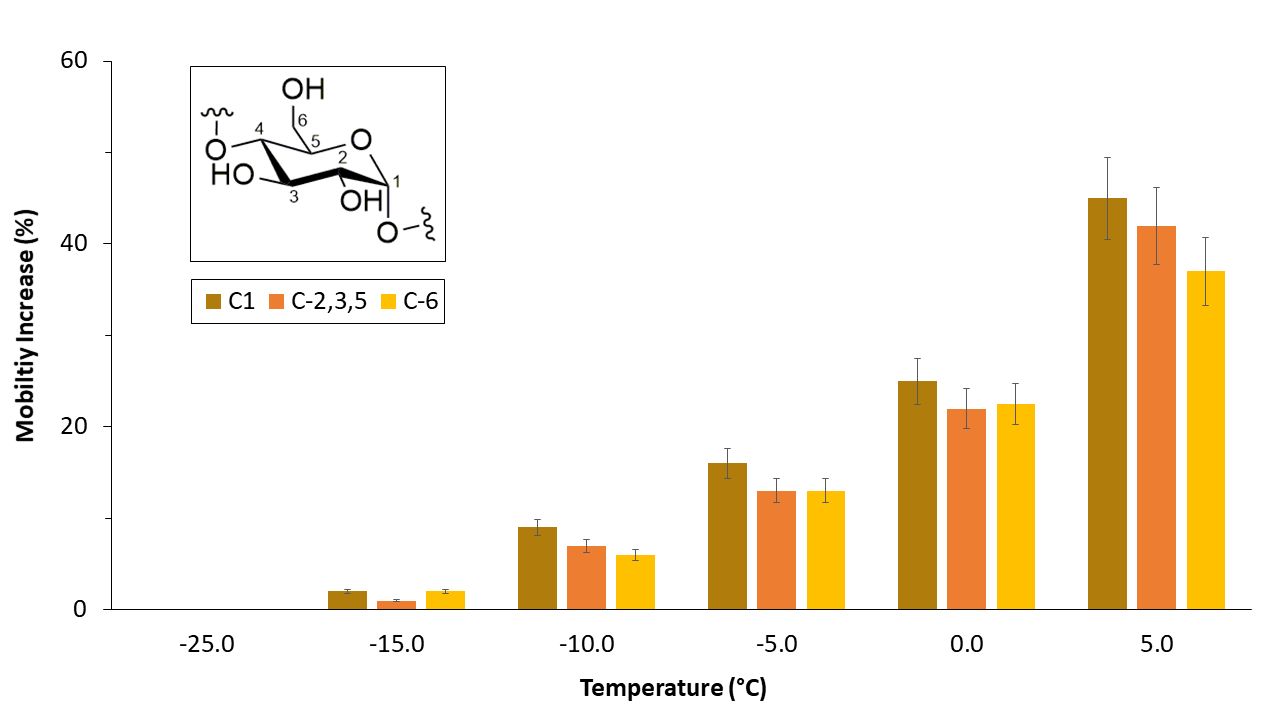


*Figure S18: Estimated average mobility levels (%) in low temperature isothermally stored normal maize hydrogels across C‑1, C‑2,3,5 and C‑6 atomic regions, as a function of temperature. All experiments were acquired with a minimum of 2000 scans. Error bars based on the S/N ratio of our spectral data.*


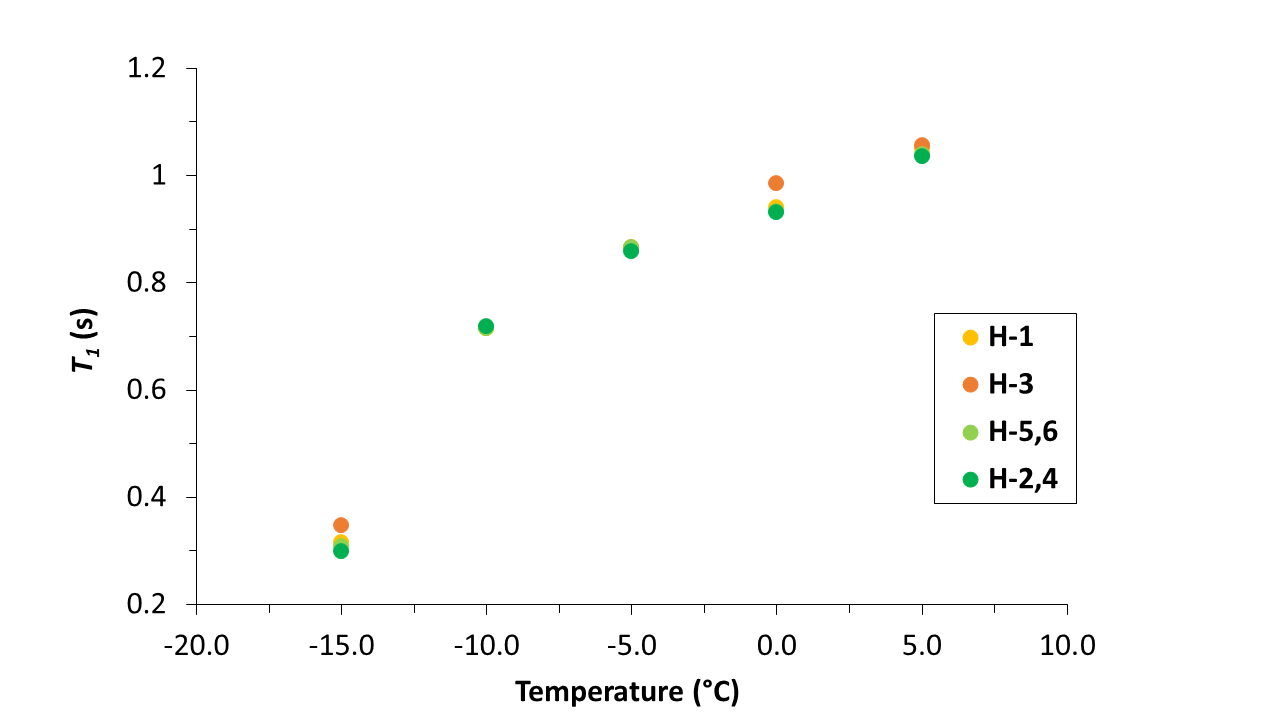


*Figure S19: ^1^H HR-MAS NMR longitudinal relaxation (T_1_) times (s) of low temperature isothermally stored normal maize starch hydrogels in the range of -15.0 to 5.0 °C.*

*Table S7: ^1^H HR-MAS NMR T_1_ times (s) of low temperature isothermally stored maize starch hydrogels measured at 5 °C.*

| Starch Hydrogel | HDO *T_1_* (s) |
| --- | --- |
| *Waxy Maize* | 1.352 |
| *Normal Maize* | 1.570 |
| *Amylomaize* | 1.283 |
| *Hylon VII™* | 1.350 |
| *Hi-Maize 260™* | 1.199 |

| 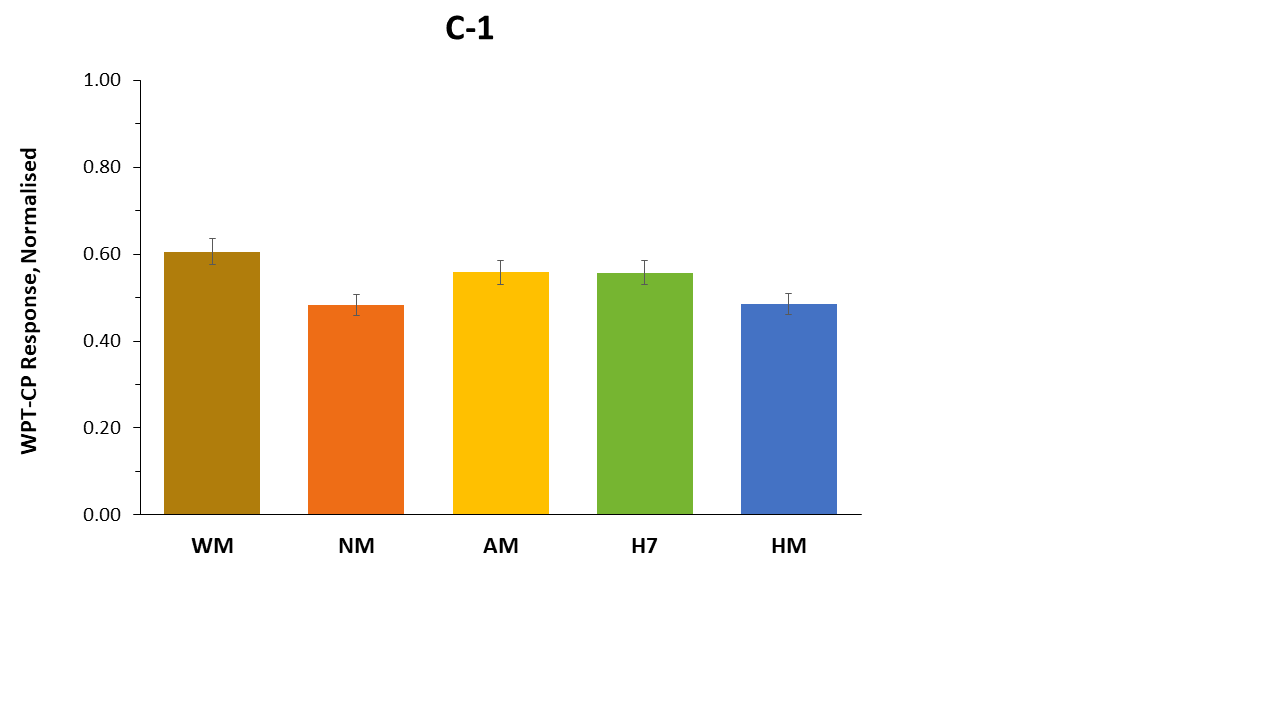 |
| --- |
| 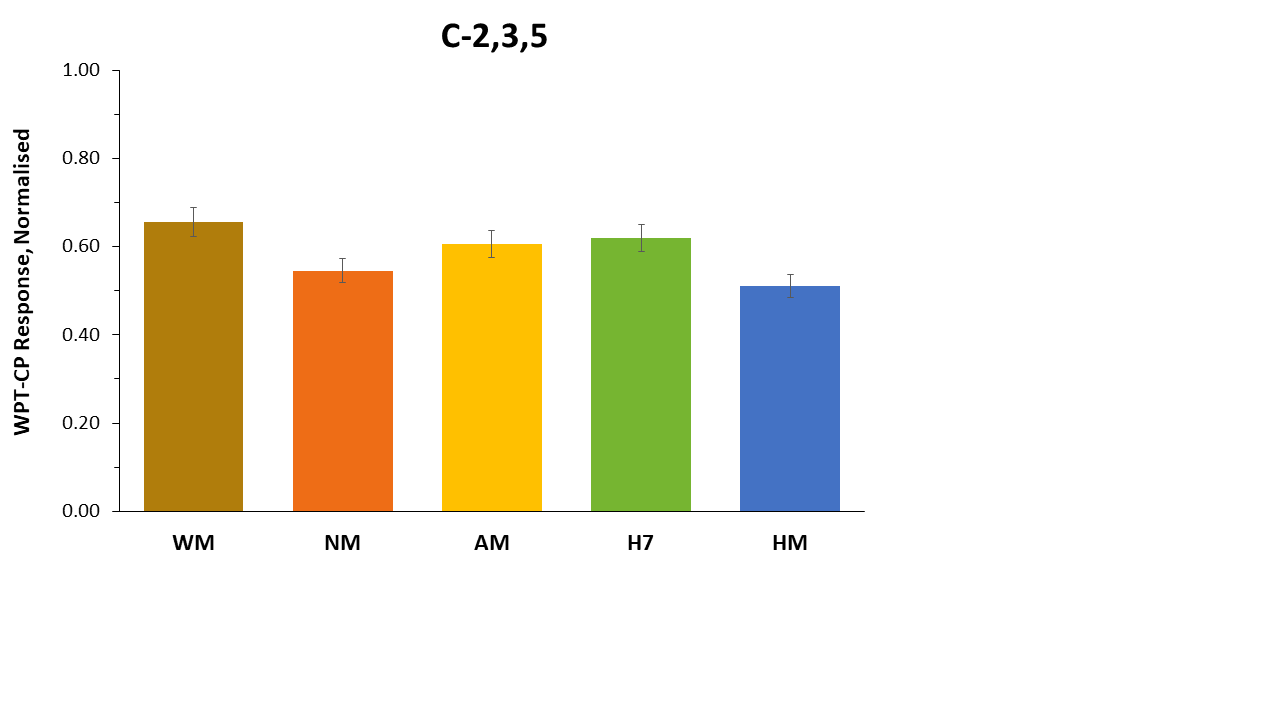 |
| 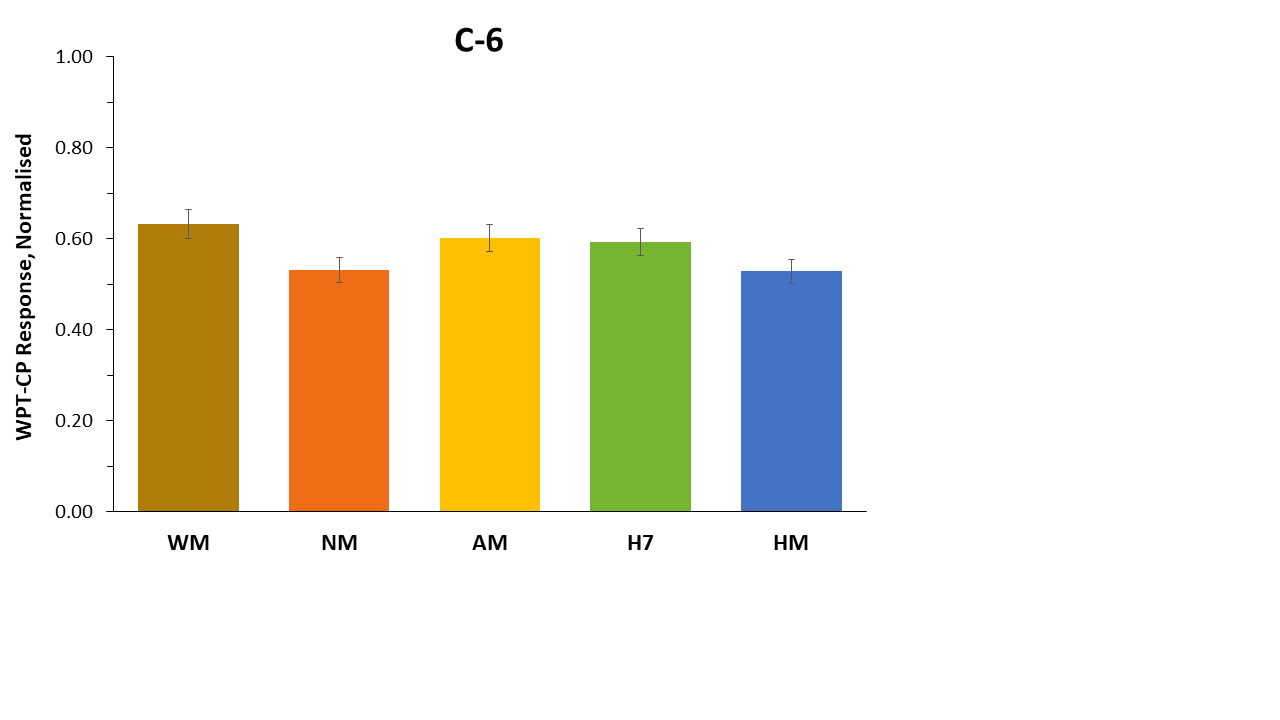 |

*Figure S20: WPT-CP response of all waxy maize (WM), normal maize (NM), amylomaize (AM), Hylon VII™ (H7) and Hi-Maize 260™ (HM) low isothermal hydrogels, separated per nuclei (C-1, C-2,3,5 and C-6), at 25 ms mixing time. The C-4 peak was disregarded in this analysis, due to its low intensity. All experiments were run at 5 °C and acquired with a minimum of 6000 scans. Error bars based on the S/N ratio in the NMR spectra data.*


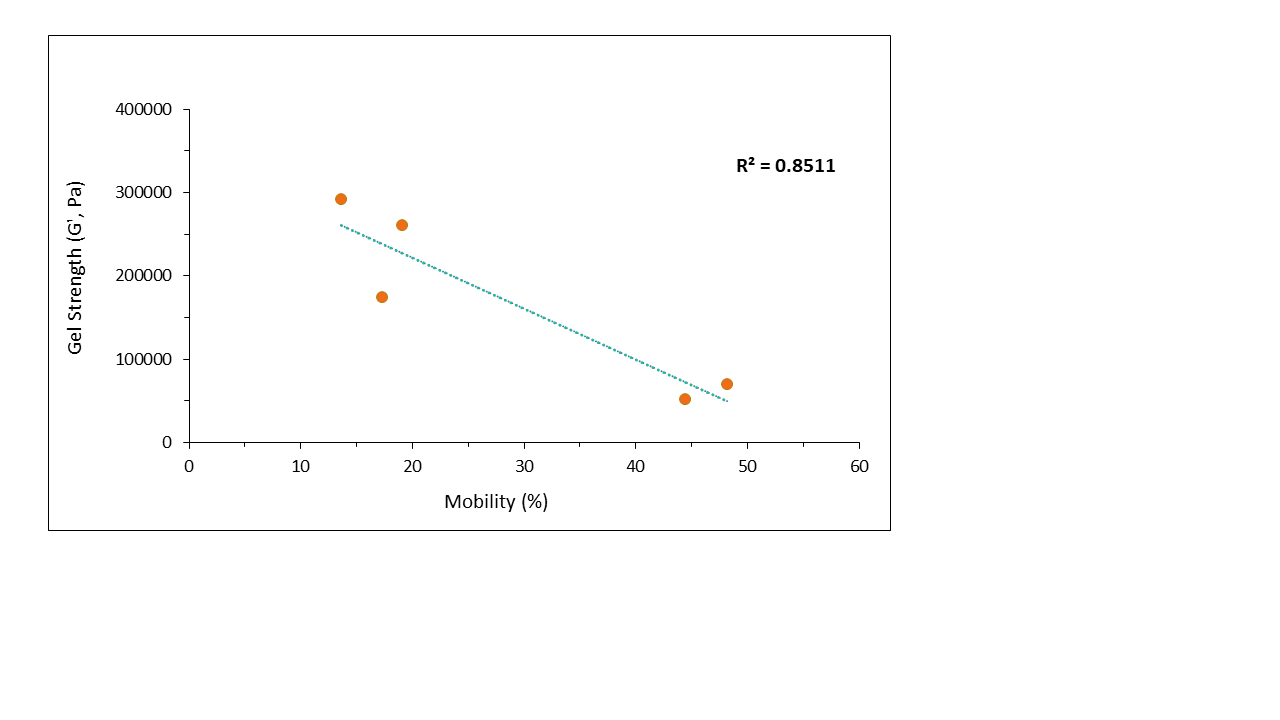


*Figure S21: Linear correlation plot between maize starch hydrogels’ strength (G’, Pa) and estimated local structural mobility levels (%), with displayed trendline and R^2^ value.*

| 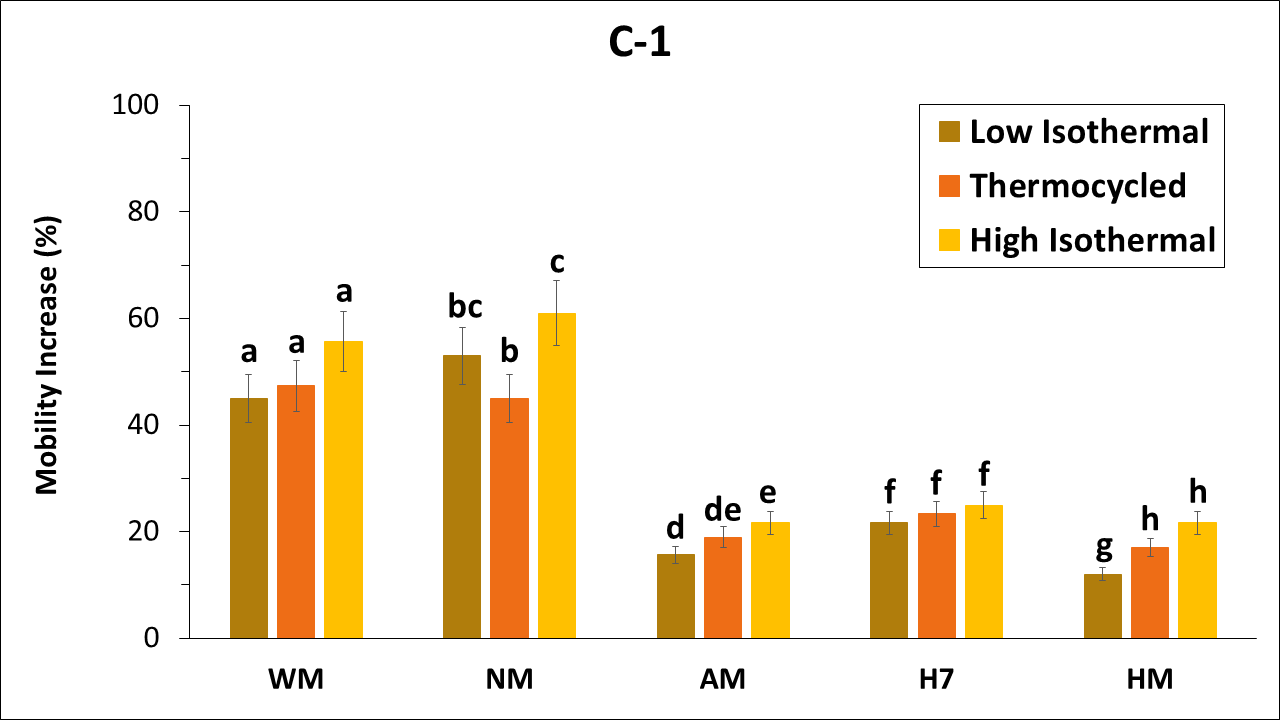 |
| --- |
| 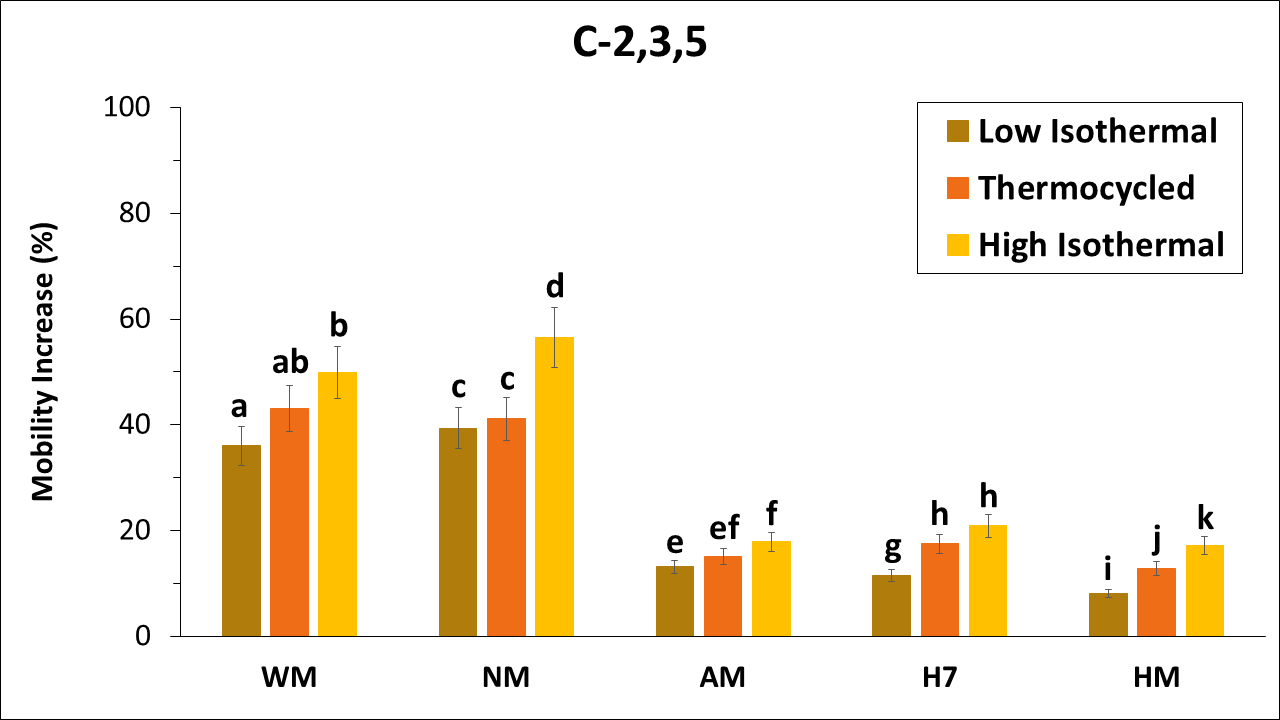 |
| 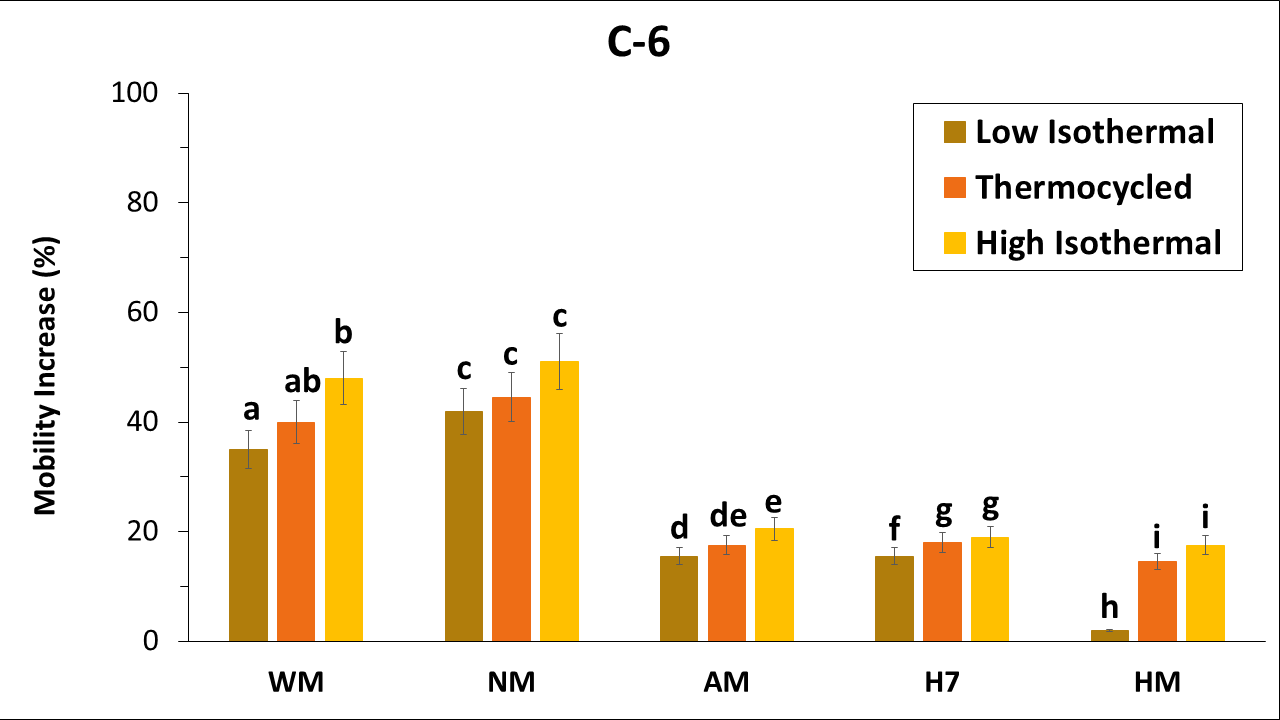 |

*Figure S22: Overall increase in local structural mobility in waxy maize (WM), normal maize (NM), amylomaize (AM), Hylon VII™ (H7) and Hi‑Maize 260™ (HM) starch hydrogels, organised by ^13^C atomic environment with C‑1 – top, C‑2,3,5 – middle and C‑6 – bottom. Error bars based on average signal to noise ratio (S/N) in our NMR spectra.*

1. **Statistical Analyses**

*Table S8: Statistical analysis of the levels of estimated structural mobility per nuclear environment in low isothermal, thermocycled and high isothermal waxy maize starch hydrogels, where different letters indicate statistically significant differences. SD values are based on the S/N ratio in the NMR spectra.*

*Table S9: Statistical analysis of the levels of estimated structural mobility per nuclear environment in low isothermal, thermocycled and high isothermal normal maize starch hydrogels, where different letters indicate statistically significant differences. SD values are based on the S/N ratio in the NMR spectra.*

*Table S10: Statistical analysis of the levels of estimated structural mobility per nuclear environment in low isothermal, thermocycled and high isothermal amylomaize starch hydrogels, where different letters indicate statistically significant differences. SD values are based on the S/N ratio in the NMR spectra.*

*Table S11: Statistical analysis of the levels of estimated structural mobility per nuclear environment in low isothermal, thermocycled and high isothermal Hylon VII® maize starch hydrogels, where different letters indicate statistically significant differences. SD values are based on the S/N ratio in the NMR spectra.*

*Table S12: Statistical analysis of the levels of estimated structural mobility per nuclear environment in low isothermal, thermocycled and high isothermal Hi-Maize 260® starch hydrogels, where different letters indicate statistically significant differences. SD values are based on the S/N ratio in the NMR spectra.*

*Table S13: Statistical analysis of the WPT-CP response of all starch hydrogels at 25 ms mixing time, where different letters indicate statistically significant differences. SD values are based on the S/N ratio in the NMR spectra.*

**References**

Gidley, M. J. (1985). Quantification of the structural features of starch polysaccharides by n.m.r. spectroscopy. *Carbohydrate Research*, *139*(C), 85–93. https://doi.org/10.1016/0008-6215(85)90009-6

Goesaert, H., Brijs, K., Veraverbeke, W. S., Courtin, C. M., Gebruers, K., & Delcour, J. A. (2005). Wheat flour constituents: How they impact bread quality, and how to impact their functionality. *Trends in Food Science and Technology*, *16*(1–3), 12–30. https://doi.org/10.1016/j.tifs.2004.02.011

Yu, L., & Christie, G. (2005). Microstructure and mechanical properties of orientated thermoplastic starches. *Journal of Materials Science*, *40*(1), 111–116.
